# Exposure to volatile organic compounds increases the risk of sarcopenia: Insights into association and mechanism

**DOI:** 10.1371/journal.pone.0335660

**Published:** 2025-10-31

**Authors:** Maosheng Yang, Hanbin Wang, Wenlong Huang, Yi Li

**Affiliations:** Department of Joint Surgery and Sports Medicine, Shandong Provincial Hospital Affiliated to Shandong First Medical University, Jinan, China; City College of New York, UNITED STATES OF AMERICA

## Abstract

**Background:**

Volatile organic compounds (VOCs) are ubiquitous in our environment, and their associations with sarcopenia are unknown. The aim of this study is to evaluate the association between VOCs exposure and sarcopenia.

**Methods:**

Data from 2429 U.S. adults (aged ≥ 20 years) were extracted from the NHANES (2011–2018). Logistic regression, LASSO, Weighted Quantile Sum (WQS) and Bayesian Kernel Machine regression (BKMR) analyses were used to assess the associations between VOCs and sarcopenia. Mediation analysis tested roles of inflammation and oxidative stress in this association. The underlying mechanisms were further investigated through database enrichment analysis, molecular docking, and molecular dynamics simulations.

**Results:**

Among the 2429 adults included, 1213 (49.9%) were male, 1216 (50.1%) were female, and the median age was 39 years (interquartile range, 29–49 years), with a prevalence of sarcopenia of 8.03%. According to the logistic analysis, nine mVOCs were significantly associated with sarcopenia, with N-acetyl-S-(3,4-dihydroxybutyl)-L-cysteine (DHBMA) identified as a potential independent risk factor (odds ratio [OR], 4.51 [95% CI: 1.7–12.1]). WQS analysis revealed a positive association between LASSO-selected 12 mVOCs and sarcopenia (OR 1.39 [95% CI: 1.17–1.90]). BKMR analysis further confirmed this association, with DHBMA showing a significant contribution. Mediation analysis confirmed that inflammation and oxidative stress exert mediating effects. GO and KEGG enrichment analyses indicated that its effects are exerted through the TNF and PI3K–Akt signaling pathways, and that DHBMA binds stably to AKT1.

**Conclusion:**

This nationally representative cross-sectional study revealed a positive correlation between exposure to mVOCs and sarcopenia via TNF and PI3K–Akt signaling pathways. DHBMA plays a potentially pivotal role in this association.

## 1. Introduction

Sarcopenia, a progressive disorder affecting skeletal muscle, is characterized by reduced muscle quality, quantity, and strength. The pooled incidence of sarcopenia in community-dwelling older adults was estimated at 12.9% (95% CI: 9.9–15.5) [1]. Prevalence rates are even higher in medical settings [2]. Patients with sarcopenia are at increased risk of functional decline, falls, fractures, mortality and hospitalization, with prolonged hospital stays and high healthcare costs [3–6]. The etiology of sarcopenia remains partially understood, although numerous studies have elucidated potential contributing factors. To date, age, sex, physical inactivity, and chronic diseases such as type 2 diabetes and cardiovascular disease are established risk factors for sarcopenia [7–9]. More recently, environmental pollutants, including heavy metals, solid fuel, PM10, and NO_2_, have been identified as significant contributors to sarcopenia [10–12].

Volatile organic compounds (VOCs) are a class of low-molecular-weight carbon-based compounds that are ubiquitous in our environment [13]. These compounds originate from natural and human-made sources, such as vehicle emissions, industrial processes, cigarettes, building materials, insecticides, and personal care products [14,15]. Numerous studies have linked VOCs exposure to various diseases. For example, VOC exposure has been shown to adversely affect adolescent growth and bone density [16]. Wu et al. reported that exposure to multiple VOCs increases the risk of kidney stones in the U.S. population [17]. Furthermore, research has highlighted the associations between VOCs exposure and cardiovascular disease, respiratory illnesses, cancer, and hearing loss, indicating that VOCs exposure poses significant health risks [18–21]. Additionally, exposure to VOCs induces oxidative stress and inflammatory responses in the body [22–24], which play key roles in sarcopenia. Oxidative stress impairs mitochondrial function, leading to cellular apoptosis and contributing to the development of sarcopenia [25]. Inflammatory mediators, such as tumor necrosis factor-α, interleukin-6, nuclear factor kappa B, and C-reactive protein, promote muscle protein breakdown and disrupt reactive oxygen species production, resulting in mitochondrial damage [26,27]. Therefore, exposure to VOCs may be associated with sarcopenia.

Owing to their high volatility, low water solubility, and rapid metabolism, direct measurement of VOCs in the blood or urine in their original form is challenging. However, their urinary metabolites can be detected for longer periods than the parent VOCs in the blood [16,17]. Thus, they are increasingly recognized as reliable biomarkers of VOC exposure. Furthermore, white blood cell (WBC) counts and serum alkaline phosphatase (ALP) levels are commonly used to evaluate inflammation [28], whereas serum bilirubin and gamma-glutamyltransferase (GGT) levels are frequently used to assess oxidative stress [29,30].

In this study, we used urinary metabolites of volatile organic compounds (mVOCs) as indicators to explore the potential association between VOCs and sarcopenia. We further utilized WBC count, as well as the levels of ALP, bilirubin, and GGT, as mediating indicators to explore the mediating roles of inflammation and oxidative stress in the mixed exposure to VOCs. Network pharmacology, molecular docking, molecular dynamics simulation was also used to further explore the mechanism.

## 2. Methods

### 2.1. Study design and population

The NHANES is a cross-sectional study that assesses the health and nutritional status of the U.S. population across all age groups. From the NHANES database, in April 2024, we selected 39,156 participants from four 2-year cycles: 2011–2012, 2013–2014, 2015–2016, and 2017–2018. The personal data of all participants were rigorously de-identified to ensure complete confidentiality. Participants aged <20 years (n = 16,539) and those with missing data on mVOCs (n = 16714), DXA (n = 2911), body mass index (BMI; n = 15), covariates (n = 434), WBC count (n = 72), ALP level (n = 41), or bilirubin level (n = 1) were excluded. This resulted in a final cohort of 2429 participants (S1 Fig). This study follows the Strengthening the Reporting of Observational Studies in Epidemiology (STROBE) reporting guidelines. The NCHS Research Ethics Review Board approved the study protocol, and written informed consent was obtained from all participants prior to their involvement.

### 2.2. Determination of urinary mVOCs

Urinary mVOCs were measured via ultra-performance liquid chromatography coupled with electrospray tandem mass spectrometry. [31] Following the NHANES guidelines, values below the lower limit of detection (LLOD) were replaced by the value obtained after the LLOD was divided by the square root of 2 (LLOD/√2). To increase the reliability of the results, only mVOCs with a detection rate of >75% were included.

### 2.3. Assessment of sarcopenia

According to the guidelines set by the Foundation for the National Institutes of Health, sarcopenia is defined as a condition in which the ratio of appendicular lean mass (combined lean mass of the arms and legs) to BMI (ALM/BMI) falls below certain thresholds: < 0.512 for women and <0.789 for men [10,32].

### 2.4. Assessment of blood biomarkers

On the basis of previous studies [33,34], serum bilirubin and GGT were selected as markers for oxidative stress, whereas the WBC count and serum ALP concentration were used to assess chronic inflammation. In accordance with the NHANES Laboratory/Medical Technologists Procedures Manual, the WBC count was measured via the Beckman Coulter method. Additionally, the DxC800 system was used to measure ALP, GGT, and total bilirubin concentrations.

### 2.5. Covariates

In alignment with prior research [35,36], we included and adjusted for the following covariates: age (continuous, years), urinary creatinine level (continuous, mg/dL), sex (male or female), race/ethnicity, education level, BMI (continuous), marital status (married, widowed, divorced, separated, never married, or living with partner), family poverty income ratio (PIR; continuous), drinking status (yes or no), smoking status (yes or no), sedentary time (continuous, min), diabetes status (yes, no, or borderline), and hypertension status (yes or no). Drinking status was assessed on the basis of participants responses to the following question: “Have you had at least 12 drinks of any type of alcoholic beverage?” Participants who had smoked fewer than 100 cigarettes in their lifetime were classified as “no smokers.” Diabetes and hypertension were diagnosed on the basis of self-reported information confirmed by medical professionals.

### 2.6. Statistical analyses

The means ± standard deviations (SDs) or medians (interquartile ranges [IQRs]) were used to present the baseline characteristics of the participants. Categorical variables are presented as percentiles. Group differences were assessed by analysis of variance and chi-square (χ2) tests. To eliminate individual variations in urine excretion, we adjusted the mVOCs by calculating the ratio of mVOCs to urinary creatinine. Owing to the highly skewed distribution of the mVOCs, we applied a logarithmic transformation to obtain an approximately normal distribution. We also calculated the Spearman correlation coefficient to assess correlations between the mVOCs.

#### 2.6.1. Logistic regression.

To evaluate the specific influence of the mVOCs on the prevalence of sarcopenia, logistic regression analysis was conducted. The mVOCs data were initially treated as continuous variables and subsequently categorized into quartiles, with the first quartile (Q1) serving as the reference point for comparison. Three models were constructed: Model 1 included no covariate adjustments; Model 2 incorporated adjustments for fundamental demographic variables, including age, sex, race, education level, marital status, PIR, and BMI; based on Model 2, Model 3 further included adjustments for drinking and smoking status, diabetes, hypertension, and sedentary time.

#### 2.6.2. LASSO–Weighted Quantile Sum (WQS) regression analysis.

As mVOCs often exist as mixtures in the natural environment and Spearman correlation analysis demonstrated correlations among their metabolites, we employed LASSO regression analysis to mitigate the effects of multicollinearity among the metabolites. A 10-fold cross-validation was conducted to assess binomial deviance and identify the optimal hyperparameter lambda (λ) for the LASSO regression model. The variables selected through LASSO screening were further analyzed. WQS regression analysis has shown effectiveness in characterizing environmental mixtures [37]. Therefore, we utilized it to explore the influence of mVOCs coexposure on sarcopenia. Owing to the substantial differences in magnitude between sedentary time and other covariates, we log-transformed sedentary time. To ensure a reliable and robust estimation, we randomly assigned 40% of the samples to a training dataset and reserved 60% for validation. We conducted bootstrapping with 10,000 iterations to construct the WQS indices, using seed 2023 for randomization.

#### 2.6.3 LASSO–Bayesian Kernel Machine regression (BKMR) analysis.

The BKMR model can estimate the health effects of multipollutant mixtures via a highly nonlinear, biologically based dose–response function [38]. We conducted BKMR analysis of metabolites selected through LASSO. BKMR analysis was also used as part of the sensitivity analysis. The cumulative effect of mixed mVOCs exposure was estimated via a Markov chain Monte Carlo algorithm, with the number of iterations set to 50,000. The estimated univariate exposure–response functions were graphically represented to explore potential nonlinearities. To evaluate the specific influence of each metabolite, an exposure–response curve was constructed while maintaining other chemical exposures at constant levels at the 25th, 50th, and 75th percentiles. Previous research has identified smoking and diabetes as key factors in sarcopenia [8,39], and smoking is a significant source of VOCs exposure. A stratified analysis of individuals with varying smoking and diabetic statuses was conducted.

#### 2.6.4. Mediation analysis.

A mediation analysis was conducted to investigate the mediating role of inflammatory and oxidative stress markers in the association between mVOC exposure and sarcopenia. We employed a nonparametric bootstrapping method (n = 1000) to conduct mediation analysis, assuming that the exposure–response relationship between mVOCs (X) and sarcopenia (Y) is mediated by biomarkers (M). The proportion of an indirect effect to the total effect reflects the efficacy of the mediator.

Logistic, LASSO, BKMR, and WQS regression analyses and mediation analysis were conducted via the ‘caret,’ ‘glmnet,’ ‘bkmr,’ ‘gWQS,’ and ‘mediation’ packages, respectively, in R software (version 4.3.2). A P value of <0.05 was considered to indicate statistical significance in all two-sided tests.

#### 2.6.5. Network pharmacological analysis.

Potential targets of mVOCs were predicted based on their SMILES structures using the SEA (https://sea.bkslab.org/) and SwissTargetPrediction (http://swisstargetprediction.ch) databases, while disease targets associated with sarcopenia were retrieved by searching the keyword “sarcopenia” in the GenCards (https://www.genecards.org) and OMIM (https://www.omim.org/) databases. The Venny online tool (v2.1, https://bioinfogp.cnb.csic.es/tools/venny/) was utilized to identify the overlapping targets between mVOCs and sarcopenia. These common targets were subsequently submitted to the DAVID (https://davidbioinformatics.nih.gov) database for Gene Ontology (GO) functional enrichment analysis and Kyoto Encyclopedia of Genes and Genomes (KEGG) pathway enrichment analysis. Furthermore, the common targets were imported into the STRING (v12.0, https://cn.string-db.org) database to construct a protein-protein interaction (PPI) network (medium confidence threshold > 0.4; disconnected nodes were hidden). The resulting PPI network was then visualized and analyzed using Cytoscape software (version 3.10.2) based on the degree centrality of nodes.

#### 2.6.6. Molecular docking.

The 2D structures of mVOCs were retrieved from the PubChem database (https://pubchem.ncbi.nlm.nih.gov/). The 3D crystal structures of the target proteins were obtained from the Protein Data Bank (PDB, http://www.rcsb.org). The acquired protein structures underwent preprocessing, including removal of water molecules, elimination of co-crystallized ligands, and addition of hydrogen atoms. The molecular docking simulations were performed using AutoDock Vina (v1.5.6) and were visualized with PyMOL (v2.6.2). All docking parameters were configured with the software’s default settings.

#### 2.6.7. Molecular dynamics simulation.

Molecular dynamics simulations were performed using GROMACS 2022. The ligand was parameterized with the GAFF force field, while the protein was described using the AMBER14SB force field in combination with the TIP3P water model to construct the protein–ligand complex system. Simulations were conducted under periodic boundary conditions in the NVT and NPT ensembles. During the simulations, all bonds involving hydrogens were constrained with the LINCS algorithm, allowing a 2 fs integration step. Long-range electrostatic interactions were calculated with the particle mesh Ewald (PME) method using a 1.2 nm cutoff, and van der Waals interactions were treated with a 10 Å cutoff, updated every 10 steps. System temperature was maintained at 298 K using the V-rescale thermostat, and pressure was controlled at 1 bar with the Berendsen barostat. After 100 ps equilibration under both NVT and NPT ensembles, a 50 ns production simulation was carried out, with snapshots saved every 10 ps. Trajectory analyses were performed with VMD and PyMOL, and binding free energies between the protein and ligand were estimated using the g_mmpbsa implementation of the MMPBSA method.

## 3. Results

### 3.1. Participant characteristics

The characteristics of the 2429 participants enrolled in this study are summarized in Table 1. The overall prevalence of sarcopenia was 8.03%. Significant differences (P < 0.05) were observed between the sarcopenia and nonsarcopenia groups in terms of age, BMI, race, education level, PIR, drinking status, hypertension status, and diabetes status. We finally selected 16 mVOCs with detection rates > 75%: 2MHA, 34MH, AAMA, AMCC, ATCA, BMA, BPMA, CEMA, CYMA, DHBMA, HPM2, 3HPMA, MADA, MHBMA3, PGA, and HPMMA. S2 Fig depicts the Spearman correlation coefficients among the 16 mVOCs. Substantial correlations (r > 0.50) were noted among various metabolites.

**Table 1:** Participant characteristics.

Variables	Total	Nonsarcopenia	Sarcopenia	P value
	(n = 2429)	(n = 2234, 91.97%)	(n = 195, 8.03%)	
Age (median [IQR])	39.00 [29.00, 49.00]	38.00 [29.00, 48.00]	46.00 [32.50, 55.00]	**<0.001**
Sex, n (%)				0.779
Male	1213 (49.9)	1118 (50.0)	95 (48.7)	
Female	1216 (50.1)	1116 (50.0)	100 (51.3)	
BMI (median [IQR])	27.80 [23.80, 32.60]	27.40 [23.60, 31.90]	33.70 [28.70, 38.95]	**<0.001**
Race, n (%)				**<0.001**
Mexican American	330 (13.6)	268 (12.0)	62 (31.8)	
Other Hispanic	260 (10.7)	226 (10.1)	34 (17.4)	
Non-Hispanic White	873 (35.9)	825 (36.9)	48 (24.6)	
Non-Hispanic Black	515 (21.2)	501 (22.4)	14 (7.2)	
Other races including multiracial	451 (18.6)	414 (18.5)	37 (19.0)	
Education level, n (%)				**<0.001**
Less than 9th grade	116 (4.8)	92 (4.1)	24 (12.3)	
9th–11th grade (includes 12th grade with no diploma)	276 (11.4)	252 (11.3)	24 (12.3)	
High school graduate/GED or equivalent	529 (21.8)	472 (21.1)	57 (29.2)	
Some college or AA degree	786 (32.4)	736 (32.9)	50 (25.6)	
College graduate or above	722 (29.7)	682 (30.5)	40 (20.5)	
PIR	2.20 [1.11, 4.34]	2.24 [1.12, 4.39]	1.79 [1.03, 3.42]	**0.017**
Marital status, n (%)				0.138
Married	1138 (46.9)	1035 (46.3)	103 (52.8)	
Widowed	30 (1.2)	29 (1.3)	1 (0.5)	
Divorced	214 (8.8)	194 (8.7)	20 (10.3)	
Separated	74 (3.0)	65 (2.9)	9 (4.6)	
Never married	684 (28.2)	640 (28.6)	44 (22.6)	
Living with partner	289 (11.9)	271 (12.1)	18 (9.2)	
Drinking status, n (%)				**0.001**
Yes	1964 (80.9)	1825 (81.7)	139 (71.3)	
No	465 (19.1)	409 (18.3)	56 (28.7)	
Smoking status, n (%)				0.65
Yes	965 (39.7)	891 (39.9)	74 (37.9)	
No	1464 (60.3)	1343 (60.1)	121 (62.1)	
Sedentary time (min)	360.00 [240.00,480.00]	360.00 [240.00,480.00]	300.00 [180.00,480.00]	0.122
Hypertension, n (%)				**0.012**
Yes	565 (23.3)	505 (22.6)	60 (30.8)	
No	1864 (76.7)	1729 (77.4)	135 (69.2)	
Diabetes, n (%)				**<0.001**
Yes	187 (7.7)	152 (6.8)	35 (17.9)	
No	2199 (90.5)	2046 (91.6)	153 (78.5)	
Borderline	43 (1.8)	36 (1.6)	7 (3.6)	

Notes: Continuous variables are presented as the median (interquartile range, IQR), whereas categorical variables are presented as numbers and percentages. BMI: body mass index, GED: General educational development, AA degree: Associate of arts degree, PIR: family poverty income ratio. The bold number indicates the p value < 0.05.

### 3.2. Logistic regression

**Fig 1:**
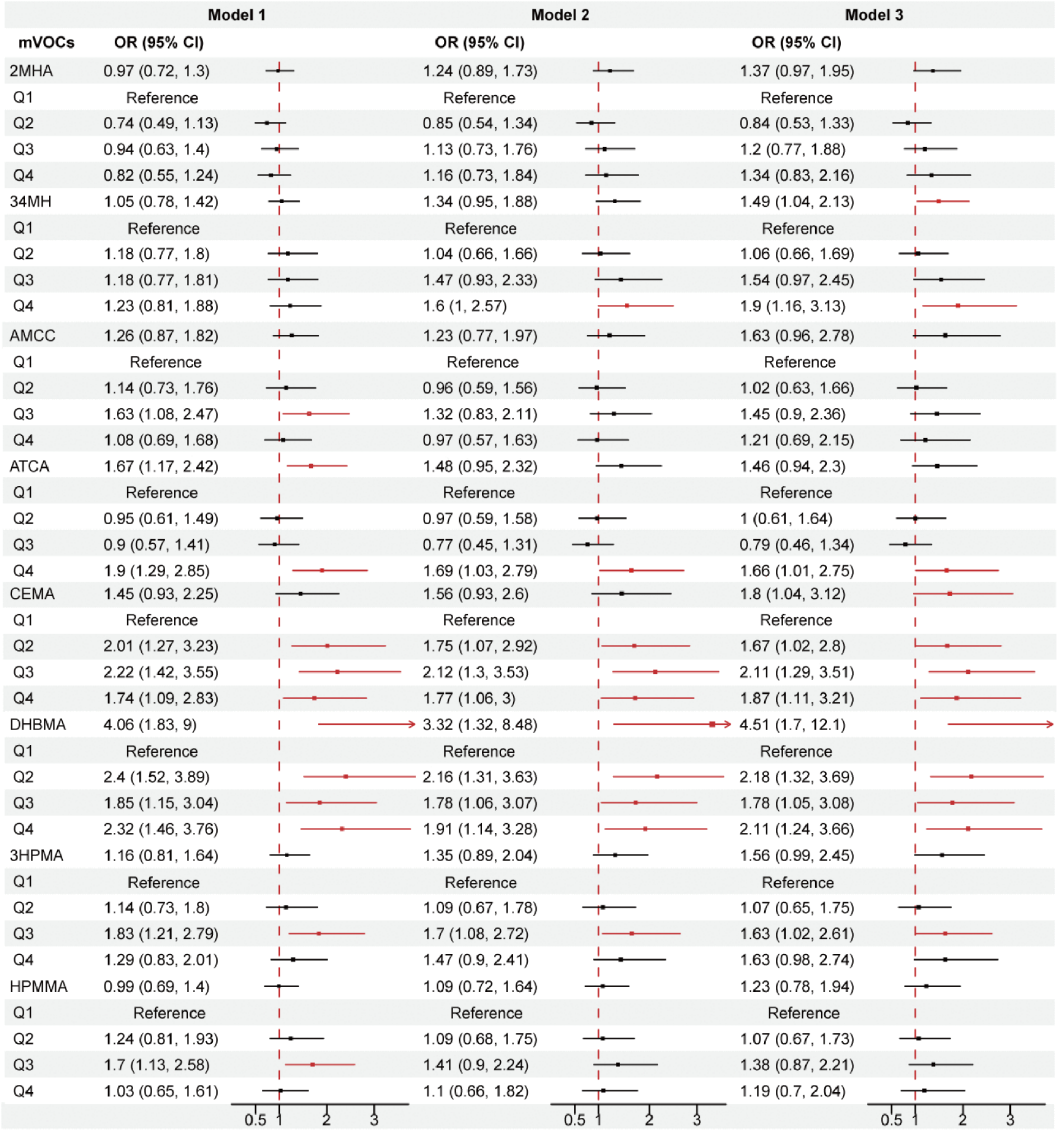
Forest plot of multivariate logistic regression analysis of mVOCs with sarcopenia. Notes: Model 1 included no covariate adjustments. Model 2 incorporated adjustments for age, sex, race, education level, marital status, PIR, and BMI. Model 3 incorporated adjustments for age, sex, race, education level, marital status, PIR, BMI, drinking and smoking status, diabetes, hypertension, and sedentary time. BMI: body mass index, PIR: family poverty income ratio. Red represents a P value less than 0.05.

We utilized univariate and multivariate regression models to assess the potential relationships between quartiles of log10-transformed mVOC concentrations and sarcopenia risk. Fig 1 and S1 Table provide the comprehensive correlation results. Notably, ATCA was significantly associated with an increased risk of sarcopenia in Q4 across Models 1, 2, and 3, with odds ratios (ORs) of 1.9, 1.69, and 1.66, respectively (all P < 0.05). CEMA also exhibited significant correlations across Q2–Q4 in all the models (P < 0.05), and a notable OR of 1.8 (P = 0.035) was obtained in the continuous variable analysis of Model 3. DHBMA demonstrated significant associations in both the continuous and categorical variable analyses (Q2–Q4) across all the models, with the highest ORs of 4.06, 3.32, and 4.51 for the continuous variable analysis of Models 1, 2, and 3, respectively (all P* *< 0.05). These findings indicate that the abovementioned pollutants significantly contribute to the onset and progression of sarcopenia.

### 3.3. LASSO–WQS regression analysis

**Fig 2:**
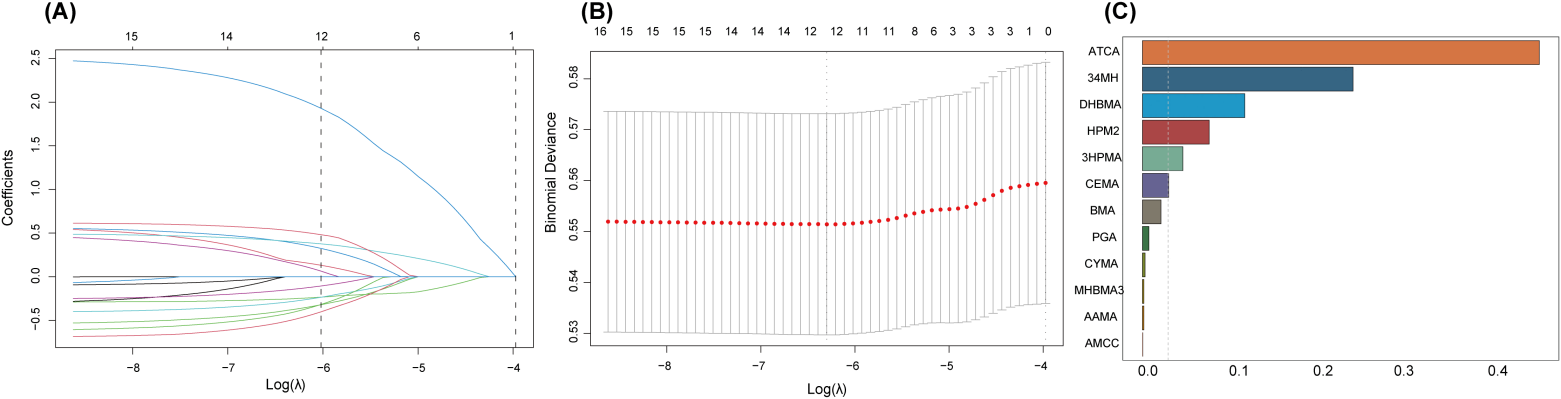
Results of LASSO and WQS regression analysis for mVOCs. Notes: The analysis incorporated adjustments for age, sex, race, education level, marital status, PIR, BMI, drinking and smoking status, diabetes, hypertension, and sedentary time. (A) Regression coefficient path diagram, where the vertical axis indicates the coefficient values and the lower horizontal axis represents ln(λ). (B) Cross-validation curve based on LASSO regression analysis, with the horizontal axis showing the logarithm of the penalty coefficient (ln λ) and the vertical axis displaying the binomial deviance. (C) Index weights from WQS regression analysis for each mVOC associated with sarcopenia.

To mitigate the effects of multicollinearity, LASSO regression analysis was performed to select 12 mVOCs (34MH, AAMA, AMCC, ATCA, BMA, CEMA, CYMA, DHBMA, HPM2, 3HPMA, MHBMA3, and PGA), as they were strongly associated with the risk of sarcopenia. S2 Table shows the strongest association between DHBMA and sarcopenia based on the regression model, as evidenced by its coefficient of 0.145. Fig 2A shows the number of nonzero coefficients for various λ values in the model. Fig 2B illustrates the relationship between binomial deviance and the ln-transformed λ value based on LASSO regression analysis. After 10-fold cross-validation, the optimal λ value with minimum binomial deviance was determined to be 0.001838 (ln-transformed λ = −6.30). In the WQS regression analysis, mixed exposure to mVOCs was significantly associated with an increased risk of sarcopenia, with an OR of 1.39 (95% CI: 1.17–1.90, P = 0.04). Fig 2C shows the mVOCs contributing to sarcopenia, with ATCA showing the highest contribution (0.454), followed by 34MH (0.241) and DHBMA (0.117).

### 3.4. LASSO–BKMR regression analysis

**Fig 3:**
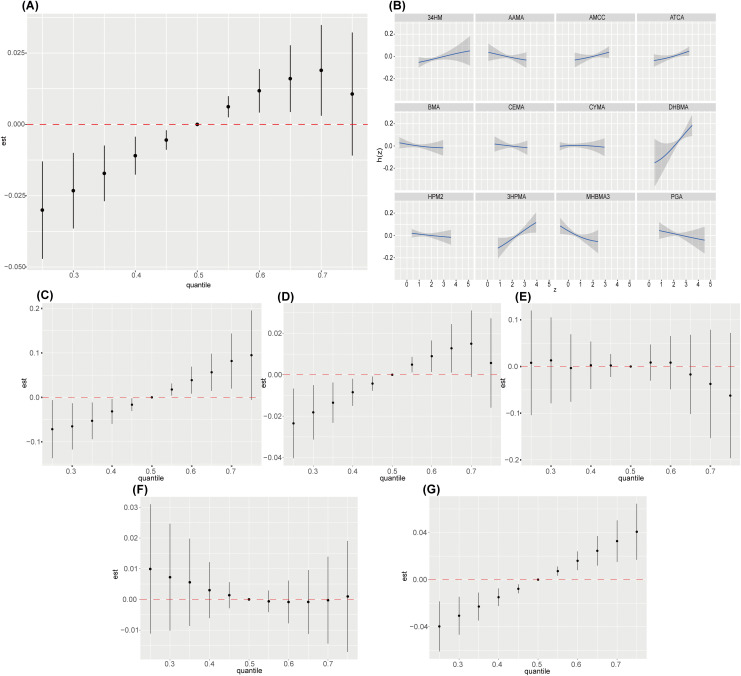
Associations between mVOCs and sarcopenia assessed by the BKMR model. Notes: The analysis incorporated adjustments for age, sex, race, education level, marital status, PIR, BMI, drinking and smoking status, diabetes, hypertension, and sedentary time. (A) Impact of mixed exposure to mVOCs on sarcopenia. (B) Univariate exposure–response functions between the screened mVOCs and sarcopenia. (C) Impact of mixed exposure to mVOCs on sarcopenia in diabetic populations. (D) Impact of mixed exposure to mVOCs on sarcopenia in nondiabetic populations. (E) Impact of mixed exposure to mVOCs on sarcopenia in populations with borderline diabetes. (F) Impact of mixed exposure to mVOCs on sarcopenia in smoking populations. (G) Impact of mixed exposure to mVOCs on sarcopenia in nonsmoking populations.

BKMR regression was employed to assess the combined influence of the 12 mVOCs selected through LASSO regression on the risk of sarcopenia comprehensively. It was also used for sensitivity analysis. Fig 3A depicts the changes in sarcopenia risk at various percentiles of mixed mVOC exposure, with the 50th percentile risk used as a reference. We observed a significant positive correlation between mixed mVOC exposure and sarcopenia. Fig 3B shows the univariate exposure–response relationships while maintaining the concentrations of other mVOCs at their median levels. Significant positive correlations were observed between 34MH, DHBMA, ATCA, and 3HPMA and sarcopenia, whereas MHBMA3 and PGA exhibited a distinctly negative association. No nonlinear associations were observed. The posterior inclusion probabilities (PIPs) are listed in S3 Table. Among the mVOCs, DHBMA had the greatest contribution to the combined effect (PIP: 0.916), followed by 3HPMA, MHBMA3, ATCA, CYMA and 34MH, with PIPs of 0.829, 0.786, 0.679, 0.534, and 0.502, respectively. As shown in S3 Fig, DHBMA and 3HPMA significantly increased the risk of sarcopenia when other mVOC concentrations were maintained at constant levels at the 25th, 50th, and 75th percentiles. Conversely, MHBMA3 negatively influences sarcopenia, particularly at the 50th and 75th percentiles. As shown in S3 Fig, no significant interactions were observed among the mVOCs.

To further investigate the role of mVOCs across different populations, we conducted a subgroup analysis on the basis of smoking status (smokers: n = 965; nonsmokers: n = 1464) and diabetic status (diabetic group: n = 187; nondiabetic group: n = 2199; borderline diabetes: n = 43). Figs 3G, 3C and 3D show a significant positive correlation between mVOCs exposure and sarcopenia in the nonsmoker, diabetic, and nondiabetic groups. However, Fig 3F and 3E indicate no significant link between mVOCs exposure and sarcopenia in smokers or individuals with borderline diabetes. Furthermore, as depicted in Fig 3C and 3D, the impact of mVOCs exposure on sarcopenia was more pronounced in the diabetic group than in the nondiabetic group.

### 3.5. Mediation analysis

**Fig 4:**
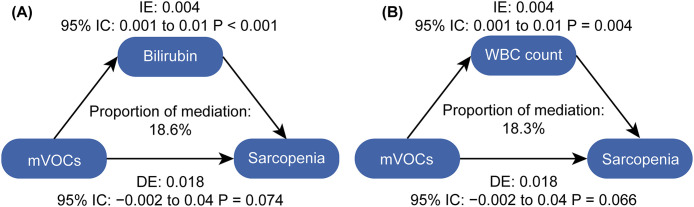
Mediation analysis of inflammatory and oxidative stress in the association between VOC exposure and sarcopenia. Notes: The analysis incorporated adjustments for age, sex, race, education level, marital status, PIR, BMI, drinking and smoking status, diabetes, hypertension, and sedentary time. (A) Mediation analysis of bilirubin in the association between mVOCs and sarcopenia. (B) Mediation analysis of WBC count in the association between mVOCs and sarcopenia. WBC, white blood cell; mVOCs, metabolites of volatile organic compounds; IE, indirect effect; DE, direct effect.

We utilized the ‘mediation’ package in R to examine the mediating effects of inflammation and oxidative stress on the association between mVOCs exposure and the risk of sarcopenia. Fig 4 illustrates significant mediation effects (P < 0.05) for the WBC count and bilirubin level, which contributed 18.3% and 18.6%, respectively, to the link between mVOC exposure and increased sarcopenia risk. No significant mediation effects were observed for ALP or GGT levels (S4 Fig).

### 3.6. Target Identification of mVOCs

**Fig 5:**
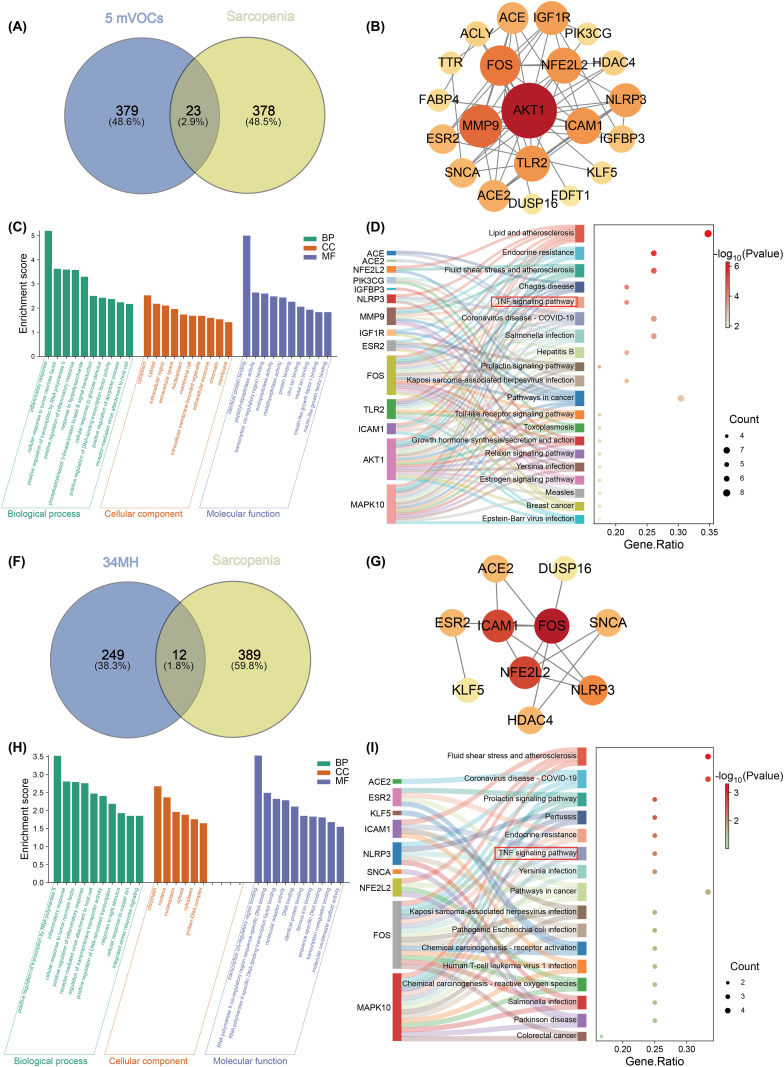
Combined network pharmacology analysis of ATCA, 34MH, DHBMA, HPM2, and 3HPMA, and an individual analysis of 34MH. Notes: (A) Overlapping targets between the five mVOCs and sarcopenia. (B) PPI network of the 23 overlapping targets. (C) Top 10 GO enrichment terms of the 23 overlapping targets, including biological process (BP), cellular component (CC), and molecular function (MF) (P < 0.05). (D) Top 20 KEGG pathways of the 23 overlapping targets (P < 0.05). (F) Overlapping targets between 34MH and sarcopenia. (G) PPI network of the 12 overlapping targets. (H) Top 10 GO enrichment terms of the 12 overlapping targets, including BP, CC, and MF (P < 0.05). (I) Top 20 KEGG pathways of the 12 overlapping targets (P < 0.05).

**Fig 6:**
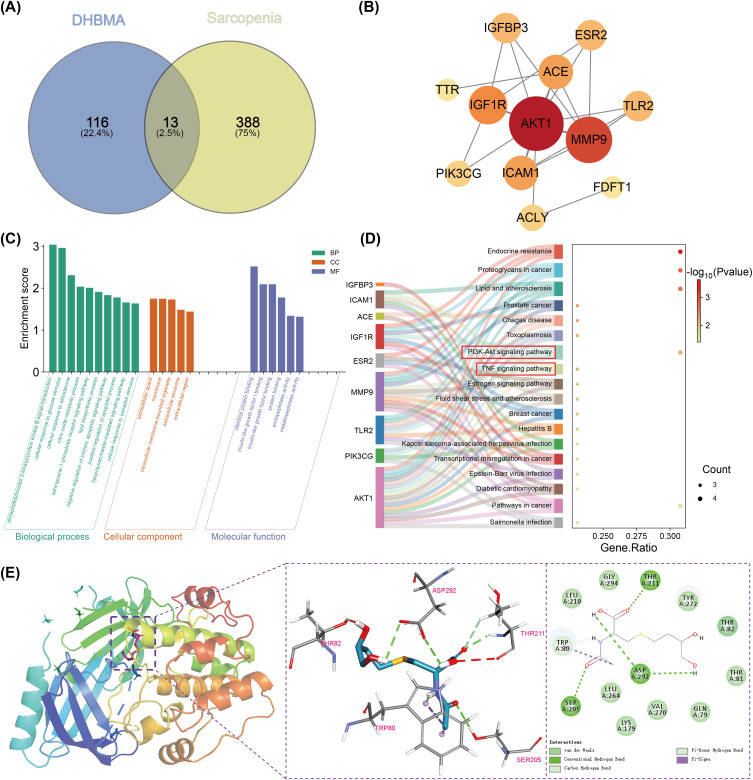
Network pharmacology analysis of DHBMA. Notes: (A) Overlapping targets between the DHBMA and sarcopenia. (B) PPI network of the 13 overlapping targets. (C) Top 10 GO enrichment terms of the 13 overlapping targets, including BP, CC, and MF (P < 0.05). (D) Top 20 KEGG pathways of the 13 overlapping targets (P < 0.05). (E) Molecular docking analysis of DHBMA with AKT1.

Guided by WQS and BKMR results, five mVOCs—ATCA, 34MH, DHBMA, HPM2, and 3HPMA—were selected for in-depth mechanistic analysis. The SMILES strings of each compound were submitted to the SEA and SwissTargetPrediction to predict putative targets. Using “sarcopenia” as a query, 401 related genes were retrieved from GeneCards and OMIM; Venny overlap analysis yielded 23 shared targets for the five-mVOC combination and 1 (ATCA), 13 (DHBMA), 12 (HPM2), 12 (34MH), and 11 (3HPMA) targets for each individual compound (Figs 5A and 5F, S4 File). Overlapping genes were subjected to GO and KEGG enrichment analysis via DAVID and to PPI analysis via STRING. The five-mVOC set was enriched for inflammatory response GO terms and KEGG TNF signaling, with STRING PPI implicating AKT1, MMP9, FOS, ICAM1, and NLRP3 (Figs 5B, 5C and 5D). 34MH produced enrichment and PPI profiles similar to the combined set (Figs 5G, 5H and 5I). DHBMA was associated with both inflammation-related and phosphorylation-related GO terms; KEGG enrichment included TNF and PI3K–Akt pathways, and PPI analysis highlighted phosphorylation- and inflammation-related nodes (Fig 6). ATCA had only one overlapping gene and could not be meaningfully analyzed; HPM2 and 3HPMA returned significant enrichments (P < 0.05) that were not related to sarcopenia. Collectively, these results indicate that 34MH and DHBMA are likely the principal drivers of the observed associations.

### 3.7. DHBMA-AKT1 Binding Stability Validation

Both the combined five-mVOC and DHBMA specific PPI networks nominated AKT1 as a putative hub target. Consequently, DHBMA was docked to AKT1 and the top-ranked complex was used for molecular dynamics simulations. In the docked pose DHBMA formed hydrogen bonds with Thr211, Asp292, and Ser205; a π–σ (pi–sigma) hydrophobic contact with Thr80; and van der Waals contacts with Leu210, Lys179, and Thr81 (Fig 6E). The predicted binding energy was −6.1 kcal/mol, consistent with a stable binding mode.

**Fig 7:**
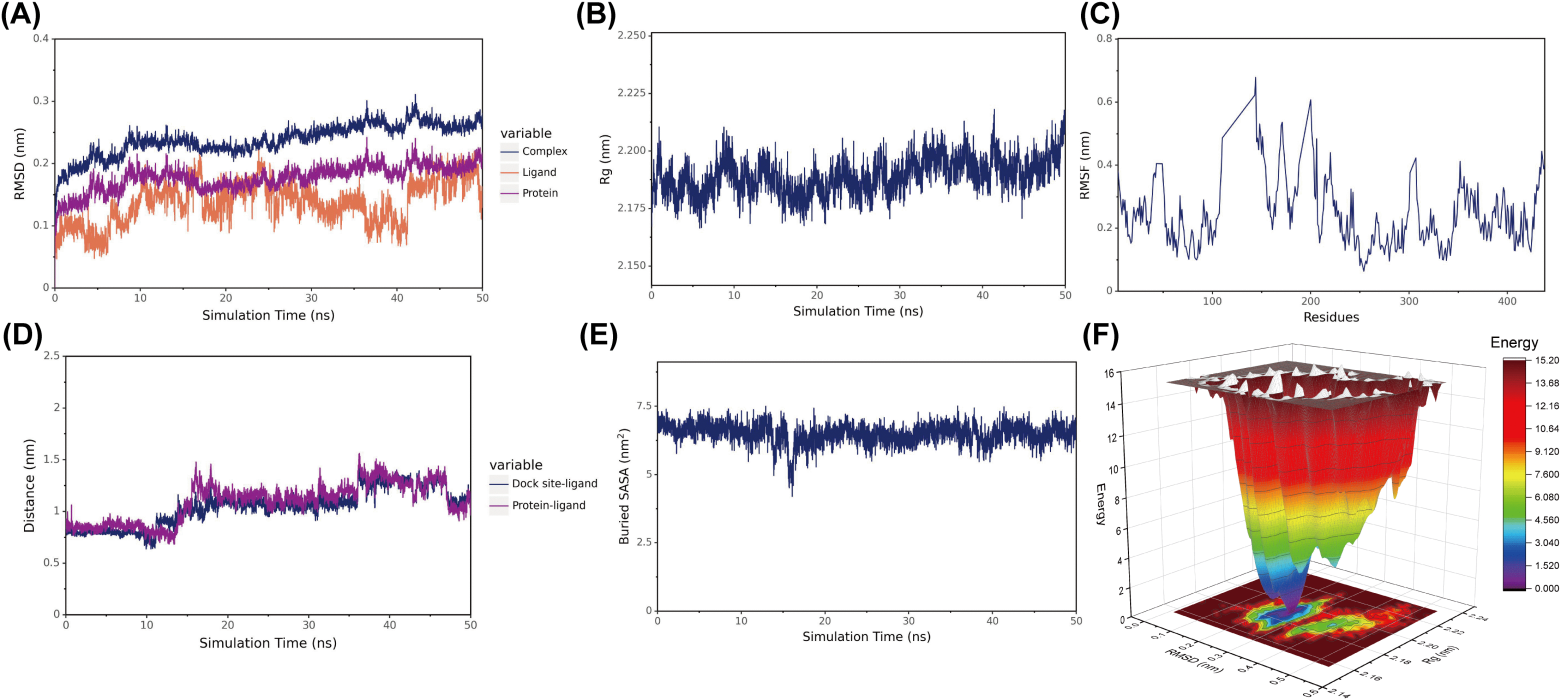
Molecular dynamics simulation of the DHBMA-AKT1 complex. Notes: (A) Root mean square deviation (RMSD) of the protein (AKT1), ligand (DHBMA), and the complex. (B) Radius of gyration (Rg) of the complex. (C) Root mean square fluctuation (RMSF) per residue of AKT1. (D) Distance between the ligand centroid and the binding pocket center. (E) Buried solvent accessible surface area (Buried SASA) between DHBMA and AKT1. (F) Free energy landscape analysis.

The molecular dynamics simulations indicated stable binding of the complex. The root-mean-square deviation (RMSD) of the protein fluctuated around 0.2 nm, suggesting the structure reached equilibrium (Fig 7A). The radius of gyration (Rg) remained stable within a certain range, reflecting a consistent overall compactness of the complex (Fig 7B). The distance from the DHBMA centroid to the geometric center of the Akt1’s binding site equilibrated, indicating that the complex reached a stable binding conformation (Fig 7D). Concurrently, the buried solvent accessible surface area (Buried SASA) analysis showed a converging trend, indicating that the contact surface between the ligand and the protein became stable (Fig 7E). Furthermore, the free energy landscape analysis revealed a deep, narrow energy well, confirming the overall structural stability of the complex (Fig 7F).

## 4. Discussion

This study investigated the association between VOCs exposure and an increased risk of sarcopenia via standard logistic regression analysis, along with WQS and BKMR models, following variable selection through LASSO regression. Additionally, we conducted a mediation analysis to explore the role of inflammation and oxidative stress markers in mediating the abovementioned associations, followed by further mechanistic investigation through network pharmacology, molecular docking, and molecular dynamics simulation. The key findings were as follows: (1) Logistic regression, WQS regression and BKMR analysis revealed a significant positive correlation between these pollutants and sarcopenia. (2) Mediation analysis revealed that the inflammation and oxidative stress potentially mediate the link between VOCs exposure and increased sarcopenia risk. (3) Network pharmacology suggested that 34MH and DHBMA exert their effects through the TNF signaling pathway. Complementary molecular docking and dynamics simulations demonstrated stable binding of DHBMA to AKT1, suggesting a potential role in regulating the PI3K/AKT pathway.

Prior studies have demonstrated that sarcopenia contributes to functional decline, falls, and increased mortality, affecting 5%–10% of individuals aged >65 years. [40] VOCs are crucial environmental pollutants, including chemicals such as benzene. [41] These substances are ubiquitous in various environments, such as homes and offices, making exposure almost unavoidable. [13] In this study, we initially applied logistic regression analysis to identify sarcopenia risk factors. Our findings indicate that the metabolites CEMA, ATCA, DHBMA, 34MH, and 3HPMA, which correspond to acrolein, cyanide, 1,3-butadiene, xylene, and acrolein, respectively, are linked to sarcopenia. Furthermore, DHBMA was identified as an independent risk factor for sarcopenia development. A study by Chen et al. demonstrated that acrolein significantly inhibits myogenic differentiation in vitro and induces muscle wasting while delaying muscle regeneration in mice. [42] Another study showed that feeding dogs cyanide-containing food leads to delayed skeletal muscle development. [43] To the best of our knowledge, no research has directly examined the link between exposure to 1,3-butadiene or xylene and sarcopenia. However, emerging evidence indicates that exposure to 1,3-butadiene diminishes mitochondrial function and elevates oxidative stress, both of which are key factors in sarcopenia development. [44,45] Salimi et al. also demonstrated that xylene causes oxidative stress and mitochondrial damage in isolated human lymphocytes. [46] The current study may offer valuable insights for future research in this area.

LASSO regression–adjusted WQS regression and BKMR analyses were used to examine the relationship between mixed mVOCs exposure and sarcopenia. Both analyses indicated that mixed mVOCs exposure substantially increased the risk of sarcopenia. Integrating WQS and BKMR analyses, five mVOCs (ATCA, 34MH, DHBMA, HPM2, and 3HPMA) were identified as key contributors, consistent with logistic regression results. To investigate the effects of VOCs exposure across different populations, we conducted stratified analyses by smoking and diabetes status. Significant associations between VOCs exposure and sarcopenia were observed in both diabetic and nondiabetic patients, with stronger associations in the diabetic group. These findings suggest an interaction between diabetes and VOCs exposure. Notably, no significant differences were detected between smokers and individuals with borderline diabetes. This may be attributed to the dominant influence of other components in cigarette smoke on sarcopenia, which could mask the effects of mVOCs exposure. Additionally, the limited sample size in the borderline diabetes group may have contributed to the nonsignificant result. Collectively, these findings substantiate that co-exposure to VOCs significantly elevates sarcopenia risk.

The mechanism linking VOCs exposure to sarcopenia remains unclear. In this study, mediation analysis confirmed that the inflammation and oxidative stress mediate this process. Exposure to VOCs can elicit inflammatory responses and oxidative stress within the body. Samadi et al. demonstrated that VOCs exposure leads to increased levels of inflammatory markers, including the WBC count. [47] Additional studies have indicated that VOCs exposure among paint workers is significantly associated with decreased total bilirubin levels. [48] Furthermore, Lee et al. reported that higher platelet and WBC counts within the normal range are independently associated with sarcopenia. [49] Wang et al. also established a positive correlation between total bilirubin levels and the appendicular skeletal muscle mass index in men. [50] Therefore, we speculated that VOCs exposure increases sarcopenia through mechanisms that promote inflammation and oxidative stress.

To further elucidate the mechanisms, network pharmacology analysis was performed on ATCA, 34MH, DHBMA, HPM2, and 3HPMA. The combined analysis indicated that these metabolites act primarily through inflammatory responses and the TNF signaling pathway, with PPI network analysis highlighting AKT1 as a key target, consistent with our mediation results. Similarly, Liu and Hong et al. reported comparable findings by mapping VOCs related targets to sarcopenia-associated genes. [51,52] This further corroborates the reliability of our preliminary statistical analyses and subsequent network pharmacology findings. Individual metabolite analyses showed that 34MH and DHBMA shared similar pathways with the combined results, whereas ATCA, HPM2, and 3HPMA yielded no significant associations, suggesting that 34MH and DHBMA play central roles in sarcopenia development. KEGG enrichment further implicated DHBMA in the PI3K–Akt signaling pathway, with PPI analysis again identifying AKT1 as a core node. Molecular docking and molecular dynamics simulations confirmed stable DHBMA–AKT1 binding, providing mechanistic evidence that VOCs may influence sarcopenia through PI3K–Akt signaling. The PI3K/Akt signaling pathway plays a critical role in promoting myogenic cell proliferation and differentiation, thereby supporting skeletal muscle development [53,54]. Dysregulation of this pathway upregulates muscle degradation markers (e.g., MuRF1, Atrogin-1, and Bnip3) while suppressing protein synthesis, contributing to the pathogenesis of sarcopenia [55]. Moreover, the PI3K/Akt pathway is closely linked to inflammatory responses, and its impairment can exacerbate inflammation and enhance oxidative stress [56]. This may represent another important pathway for VOC-associated sarcopenia.

To date, numerous diseases have been reported to be associated with mVOCs, including: chronic obstructive pulmonary disease [57], chronic kidney disease [58], cancer [59], and cardiovascular disease [60]. Our findings contribute to the evidence linking VOCs to muscle deterioration, underscoring their significance as emerging environmental risk factors for sarcopenia. From a public health perspective, these results highlight the urgent need to strengthen policies that limit VOC emissions from industrial processes, traffic, and household products, as well as to promote indoor air quality interventions. Given the pervasive nature of VOCs in daily life, strategies aimed at exposure reduction, such as improving ventilation, regulating emissions, and raising public awareness, are necessary. Clinically, these findings suggest that environmental exposure assessment should be integrated into sarcopenia risk evaluation, particularly for vulnerable populations such as older adults, individuals with obesity, and those with chronic comorbidities. Early intervention has the potential to markedly alleviate the burden of sarcopenia and mitigate its downstream consequences, including frailty, functional disability, and increased healthcare costs.

This study had several notable advantages. First, this study is the first to identify the dual involvement of TNF and PI3K/Akt signaling pathways in mediating VOC-associated sarcopenia. Second, this study employed multiple analytical approaches, including logistic regression with various models, LASSO-based WQS regression, BKMR, mediation analysis, network pharmacology, and molecular dynamics simulations, ensuring the reliability of the results. Third, by utilizing the NHANES database, we obtained an extensive sample representative of the U.S. population, enhancing the generalizability of our findings. However, this study had several limitations. First, as a cross-sectional study, a causal relationship between mVOCs exposure and sarcopenia cannot be established. Second, we excluded samples with missing data through direct deletion, which may have influenced the results. Third, although we included numerous covariates, there may be unaccounted factors that can introduce bias into the findings. Additionally, while network pharmacology and molecular dynamics simulations have provided insights into the mechanism of VOCs, these findings require confirmation through subsequent in vitro and in vivo experiments.

## 5. Conclusion

This study demonstrates that exposure to VOCs significantly increases the risk of sarcopenia. The mechanistic insights reveal that this effect is primarily mediated through the TNF signaling pathway and PI3K-Akt signaling pathway, which collectively exacerbate muscle tissue inflammation and oxidative stress, while concurrently disrupting protein synthesis, thereby driving the pathogenesis of sarcopenia.

## Supporting information

S1 Table Multivariate logistic regression analysis of log10-transformed mVOCs and their association with sarcopenia. (DOCX)

S2 Table Coefficients of the 16 mVOCs in LASSO regression analysis. (DOCX)

S3 Table PIPs of the BKMR model. (DOCX)

S1 Fig Flowchart of the participants included in this study. (DOCX)

S2 Fig Spearman correlation coefficients among the 16 mVOCs. (DOCX)

S3 Fig Association between mVOCs and sarcopenia assessed by BKMR model. (DOCX)

S4 Fig Mediation analysis of inflammatory and oxidative stress markers. (DOCX)

S2 File NHANES database raw data files for analysis. (XLSX)

S3 File Analysis code. (DOCX)

S4 File The original data of GO and KEGG analysis. (XLSX)

## Abbreviations

NHANES: National Health and Nutrition Examination Survey
VOCs: volatile organic compounds
mVOCs: volatile organic compounds
LASSO: least absolute shrinkage and selection operator
WQS: weighted quantile sum
BKMR: Bayesian kernel machine regression
OR: odds ratio
IQR: interquartile range
BMI: body mass index
GED: General educational development
AA degree: Associate of arts degree
PIR: family poverty income ratio
WBC: white blood cell
ALP: serum alkaline phosphatase
GGT: gamma-glutamyltransferase
LLOD: lower limit of detection
SD: standard deviation
IQR: interquartile range
2MHA: 2-methylhippuric acid
34MHA: 3- and 4-methylhippuric acid
AAMA: N-ace-S-(2-carbamoylethyl)-L-cysteine
AMCC: N-ace-S-(N-methylcarbamoyl)-L-cysteine
ATCA: 2-aminothiazolone-4-carboxylic acid
BMA: N-acetyl-S-(benzyl)-L-cysteine
BPMA: N-acetyl-S-(n-propyl)-L-cysteine
CEMA: N-acetyl-S-(2-carboxyethyl)-L-cysteine
CYMA: N-acetyl-S-(2-cyanoethyl)-L-cysteine
DHBMA: N-acetyl-S-(3:4-dihydroxybutyl)-L-cysteine
HPM2: N-acetyl-S-(2-hydroxypropyl)-L-cysteine
3HPMA: N-acetyl-S-(3-hydroxypropyl)-L-cysteine
MADA: mandelic acid
MHBMA3: N-acetyl-S-(4-hydroxy-2-butenyl)-L-cysteine
PGA: phenylglyoxylic acid
HPMMA: N-acetyl-S-(3-hydroxypropyl-1-methyl)-L-cysteine
PIPs: posterior inclusion probabilities
GO: Gene Ontology
KEGG: Kyoto Encyclopedia of Genes and Genomes
PPI: protein-protein interaction
BP: biological process
CC: cellular component
MF: molecular function
RMSD: Root mean square deviation
RMSF: Root mean square fluctuation
