# Supplementary material for: Exposure to volatile organic compounds increases the risk of sarcopenia: Insights into association and mechanism
Source: PLoS One. 2025 Oct 31;20(10):e0335660. doi: 10.1371/journal.pone.0335660 (PMC12578169; doi:10.1371/journal.pone.0335660)
Supplement: S1 Table — (DOCX) [file pone.0335660.s001.docx]

| **S1 Table 1. Multivariate logistic regression analysis of log10-transformed mVOCs and their association with sarcopenia.** | | | | | | |
| --- | --- | --- | --- | --- | --- | --- |
|  | **Model 1** |  | **Model 2** |  | **Model 3** |  |
|  | **OR (95% CI)** | **P value** | **OR (95% CI)** | **P value** | **OR (95% CI)** | **P value** |
| 2MHA | 0.97 (0.72, 1.3) | 0.821 | 1.24 (0.89, 1.73) | 0.206 | 1.37 (0.97, 1.95) | 0.076 |
| Q1 | Reference |  | Reference |  | Reference |  |
| Q2 | 0.74 (0.49, 1.13) | 0.167 | 0.85 (0.54, 1.34) | 0.493 | 0.84 (0.53, 1.33) | 0.452 |
| Q3 | 0.94 (0.63, 1.4) | 0.761 | 1.13 (0.73, 1.76) | 0.575 | 1.2 (0.77, 1.88) | 0.419 |
| Q4 | 0.82 (0.55, 1.24) | 0.35 | 1.16 (0.73, 1.84) | 0.521 | 1.34 (0.83, 2.16) | 0.234 |
| 34MH | 1.05 (0.78, 1.42) | 0.733 | 1.34 (0.95, 1.88) | 0.093 | 1.49 (1.04, 2.13) | **0.029** |
| Q1 | Reference |  | Reference |  | Reference |  |
| Q2 | 1.18 (0.77, 1.8) | 0.455 | 1.04 (0.66, 1.66) | 0.855 | 1.06 (0.66, 1.69) | 0.813 |
| Q3 | 1.18 (0.77, 1.81) | 0.45 | 1.47 (0.93, 2.33) | 0.103 | 1.54 (0.97, 2.45) | 0.071 |
| Q4 | 1.23 (0.81, 1.88) | 0.337 | 1.6 (1, 2.57) | **0.049** | 1.9 (1.16, 3.13) | **0.011** |
| AAMA | 0.82 (0.53, 1.26) | 0.368 | 1.01 (0.61, 1.66) | 0.981 | 1.18 (0.69, 2.02) | 0.552 |
| Q1 | Reference |  | Reference |  | Reference |  |
| Q2 | 1.24 (0.83, 1.87) | 0.298 | 1.25 (0.8, 1.95) | 0.331 | 1.27 (0.81, 2) | 0.293 |
| Q3 | 1.26 (0.84, 1.9) | 0.258 | 1.22 (0.78, 1.91) | 0.394 | 1.27 (0.81, 2.01) | 0.295 |
| Q4 | 0.77 (0.49, 1.21) | 0.26 | 0.89 (0.54, 1.47) | 0.653 | 0.99 (0.59, 1.67) | 0.98 |
| AMCC | 1.26 (0.87, 1.82) | 0.218 | 1.23 (0.77, 1.97) | 0.392 | 1.63 (0.96, 2.78) | 0.072 |
| Q1 | Reference |  | Reference |  | Reference |  |
| Q2 | 1.14 (0.73, 1.76) | 0.567 | 0.96 (0.59, 1.56) | 0.877 | 1.02 (0.63, 1.66) | 0.938 |
| Q3 | 1.63 (1.08, 2.47) | **0.02** | 1.32 (0.83, 2.11) | 0.246 | 1.45 (0.9, 2.36) | 0.125 |
| Q4 | 1.08 (0.69, 1.68) | 0.73 | 0.97 (0.57, 1.63) | 0.9 | 1.21 (0.69, 2.15) | 0.505 |
| ATCA | 1.67 (1.17, 2.42) | **0.006** | 1.48 (0.95, 2.32) | 0.086 | 1.46 (0.94, 2.3) | 0.095 |
| Q1 | Reference |  | Reference |  | Reference |  |
| Q2 | 0.95 (0.61, 1.49) | 0.825 | 0.97 (0.59, 1.58) | 0.894 | 1 (0.61, 1.64) | 0.994 |
| Q3 | 0.9 (0.57, 1.41) | 0.644 | 0.77 (0.45, 1.31) | 0.339 | 0.79 (0.46, 1.34) | 0.38 |
| Q4 | 1.9 (1.29, 2.85) | **0.001** | 1.69 (1.03, 2.79) | **0.038** | 1.66 (1.01, 2.75) | **0.048** |
| BMA | 1.04 (0.7, 1.51) | 0.851 | 1.03 (0.67, 1.57) | 0.894 | 1.03 (0.66, 1.58) | 0.889 |
| Q1 | Reference |  | Reference |  | Reference |  |
| Q2 | 0.85 (0.56, 1.27) | 0.418 | 0.81 (0.52, 1.26) | 0.357 | 0.81 (0.52, 1.27) | 0.368 |
| Q3 | 0.73 (0.47, 1.1) | 0.138 | 0.71 (0.45, 1.13) | 0.151 | 0.71 (0.45, 1.14) | 0.156 |
| Q4 | 0.94 (0.63, 1.4) | 0.768 | 0.83 (0.53, 1.29) | 0.41 | 0.83 (0.53, 1.3) | 0.417 |
| BPMA | 1.16 (0.89, 1.49) | 0.265 | 1.09 (0.81, 1.45) | 0.57 | 1.1 (0.82, 1.48) | 0.507 |
| Q1 | Reference |  | Reference |  | Reference |  |
| Q2 | 1.17 (0.77, 1.81) | 0.461 | 1.25 (0.78, 2) | 0.352 | 1.24 (0.78, 2) | 0.365 |
| Q3 | 1.38 (0.91, 2.1) | 0.133 | 1.47 (0.93, 2.35) | 0.106 | 1.47 (0.93, 2.36) | 0.105 |
| Q4 | 1.16 (0.75, 1.79) | 0.501 | 1.09 (0.67, 1.77) | 0.732 | 1.1 (0.67, 1.8) | 0.704 |
| CEMA | 1.45 (0.93, 2.25) | 0.095 | 1.56 (0.93, 2.6) | 0.09 | 1.8 (1.04, 3.12) | **0.035** |
| Q1 | Reference |  | Reference |  | Reference |  |
| Q2 | 2.01 (1.27, 3.23) | **0.003** | 1.75 (1.07, 2.92) | **0.029** | 1.67 (1.02, 2.8) | **0.046** |
| Q3 | 2.22 (1.42, 3.55) | **0.001** | 2.12 (1.3, 3.53) | **0.003** | 2.11 (1.29, 3.51) | **0.003** |
| Q4 | 1.74 (1.09, 2.83) | **0.021** | 1.77 (1.06, 3) | **0.032** | 1.87 (1.11, 3.21) | **0.021** |
| CYMA | 0.84 (0.71, 0.99) | 0.047 | 0.98 (0.81, 1.19) | 0.849 | 1.11 (0.88, 1.4) | 0.394 |
| Q1 | Reference |  | Reference |  | Reference |  |
| Q2 | 1.3 (0.87, 1.95) | 0.198 | 1.48 (0.96, 2.31) | 0.079 | 1.49 (0.96, 2.34) | 0.076 |
| Q3 | 1.11 (0.74, 1.68) | 0.612 | 1.45 (0.92, 2.28) | 0.11 | 1.51 (0.95, 2.4) | 0.082 |
| Q4 | 0.75 (0.48, 1.17) | 0.212 | 1.09 (0.65, 1.8) | 0.749 | 1.39 (0.77, 2.49) | 0.27 |

**S1 Table 1. (Continued)**

|  | **Model 1** |  | **Model 2** |  | **Model 3** |  |
| --- | --- | --- | --- | --- | --- | --- |
|  | **OR (95% CI)** | **P value** | **OR (95% CI)** | **P value** | **OR (95% CI)** | **P value** |
| DHBMA | 4.06 (1.83, 9) | **0.001** | 3.32 (1.32, 8.48) | **0.012** | 4.51 (1.7, 12.1) | **0.003** |
| Q1 | Reference |  | Reference |  | Reference |  |
| Q2 | 2.4 (1.52, 3.89) | **<0.001** | 2.16 (1.31, 3.63) | **0.003** | 2.18 (1.32, 3.69) | **0.003** |
| Q3 | 1.85 (1.15, 3.04) | **0.013** | 1.78 (1.06, 3.07) | **0.033** | 1.78 (1.05, 3.08) | **0.035** |
| Q4 | 2.32 (1.46, 3.76) | **<0.001** | 1.91 (1.14, 3.28) | **0.016** | 2.11 (1.24, 3.66) | **0.007** |
| HPM2 | 0.88 (0.6, 1.26) | 0.487 | 0.93 (0.61, 1.39) | 0.73 | 1 (0.66, 1.51) | 0.987 |
| Q1 | Reference |  | Reference |  | Reference |  |
| Q2 | 1.08 (0.73, 1.62) | 0.691 | 1.02 (0.66, 1.58) | 0.939 | 1.02 (0.66, 1.59) | 0.92 |
| Q3 | 0.89 (0.59, 1.35) | 0.597 | 1.01 (0.64, 1.58) | 0.969 | 1.07 (0.68, 1.7) | 0.765 |
| Q4 | 0.83 (0.54, 1.27) | 0.391 | 0.93 (0.59, 1.48) | 0.775 | 1.02 (0.64, 1.65) | 0.92 |
| 3HPMA | 1.16 (0.81, 1.64) | 0.405 | 1.35 (0.89, 2.04) | 0.159 | 1.56 (0.99, 2.45) | 0.052 |
| Q1 | Reference |  | Reference |  | Reference |  |
| Q2 | 1.14 (0.73, 1.8) | 0.571 | 1.09 (0.67, 1.78) | 0.738 | 1.07 (0.65, 1.75) | 0.801 |
| Q3 | 1.83 (1.21, 2.79) | **0.005** | 1.7 (1.08, 2.72) | **0.024** | 1.63 (1.02, 2.61) | **0.041** |
| Q4 | 1.29 (0.83, 2.01) | 0.264 | 1.47 (0.9, 2.41) | 0.125 | 1.63 (0.98, 2.74) | 0.06 |
| MADA | 1.06 (0.63, 1.76) | 0.829 | 1.49 (0.81, 2.69) | 0.192 | 1.72 (0.92, 3.2) | 0.086 |
| Q1 | Reference |  | Reference |  | Reference |  |
| Q2 | 1 (0.66, 1.54) | 0.987 | 0.93 (0.58, 1.48) | 0.763 | 0.97 (0.61, 1.55) | 0.899 |
| Q3 | 1.29 (0.86, 1.94) | 0.219 | 1.33 (0.86, 2.09) | 0.205 | 1.36 (0.87, 2.15) | 0.178 |
| Q4 | 0.98 (0.64, 1.5) | 0.92 | 1.27 (0.78, 2.05) | 0.337 | 1.44 (0.87, 2.37) | 0.155 |
| MHBMA3 | 0.86 (0.62, 1.17) | 0.338 | 0.99 (0.69, 1.42) | 0.96 | 1.11 (0.74, 1.65) | 0.614 |
| Q1 | Reference |  | Reference |  | Reference |  |
| Q2 | 1.11 (0.74, 1.67) | 0.628 | 1.04 (0.67, 1.63) | 0.853 | 1.01 (0.65, 1.59) | 0.95 |
| Q3 | 0.95 (0.63, 1.46) | 0.83 | 0.93 (0.59, 1.47) | 0.763 | 0.91 (0.57, 1.44) | 0.682 |
| Q4 | 1 (0.66, 1.52) | 0.993 | 1.21 (0.76, 1.94) | 0.425 | 1.42 (0.86, 2.35) | 0.173 |
| PGA | 0.98 (0.57, 1.71) | 0.954 | 1.24 (0.63, 2.39) | 0.533 | 1.41 (0.71, 2.81) | 0.328 |
| Q1 | Reference |  | Reference |  | Reference |  |
| Q2 | 1.14 (0.76, 1.72) | 0.526 | 1.03 (0.65, 1.62) | 0.915 | 1.04 (0.66, 1.65) | 0.871 |
| Q3 | 1.04 (0.69, 1.58) | 0.845 | 1.07 (0.67, 1.71) | 0.772 | 1.11 (0.69, 1.78) | 0.674 |
| Q4 | 0.98 (0.64, 1.49) | 0.914 | 1.16 (0.71, 1.9) | 0.547 | 1.3 (0.78, 2.16) | 0.32 |
| HPMMA | 0.99 (0.69, 1.4) | 0.951 | 1.09 (0.72, 1.64) | 0.688 | 1.23 (0.78, 1.94) | 0.367 |
| Q1 | Reference |  | Reference |  | Reference |  |
| Q2 | 1.24 (0.81, 1.93) | 0.326 | 1.09 (0.68, 1.75) | 0.721 | 1.07 (0.67, 1.73) | 0.774 |
| Q3 | 1.7 (1.13, 2.58) | **0.011** | 1.41 (0.9, 2.24) | 0.141 | 1.38 (0.87, 2.21) | 0.17 |
| Q4 | 1.03 (0.65, 1.61) | 0.908 | 1.1 (0.66, 1.82) | 0.724 | 1.19 (0.7, 2.04) | 0.525 |
| Notes: Model 1 included no covariate adjustments. Model 2 incorporated adjustments for age, sex, race, education level, marital status, PIR, and BMI. Model 3 incorporated adjustments for age, sex, race, education level, marital status, PIR, BMI, drinking and smoking status, diabetes, hypertension, and sedentary time. BMI: body mass index, PIR: family poverty income ratio. The bold number indicates the p value < 0.05. | | | | | | |
