# Supplementary material for: Exposure to volatile organic compounds increases the risk of sarcopenia: Insights into association and mechanism
Source: PLoS One. 2025 Oct 31;20(10):e0335660. doi: 10.1371/journal.pone.0335660 (PMC12578169; doi:10.1371/journal.pone.0335660)
Supplement: S2 Table — (DOCX) [file pone.0335660.s002.docx]

**S1 Table 2. Coefficients of the 16 mVOCs in LASSO regression analysis.**

| Variables | Coefficients |
| --- | --- |
| DHBMA | 0.145 |
| CEMA | 0.038 |
| ATCA | 0.029 |
| AMCC | 0.027 |
| 34MH | 0.012 |
| 3HPMA | 0.01 |
| BMA | −0.012 |
| CYMA | −0.015 |
| HPM2 | −0.022 |
| AAMA | −0.027 |
| PGA | −0.03 |
| MHBMA3 | −0.038 |

Notes: Analysis incorporated adjustments for age, sex, race, education level, marital status, PIR, BMI, drinking and smoking status, diabetes, hypertension, and sedentary time.
