# Supplementary material for: Exposure to volatile organic compounds increases the risk of sarcopenia: Insights into association and mechanism
Source: PLoS One. 2025 Oct 31;20(10):e0335660. doi: 10.1371/journal.pone.0335660 (PMC12578169; doi:10.1371/journal.pone.0335660)
Supplement: S3 Table — (DOCX) [file pone.0335660.s003.docx]

**S1 Table 3. PIPs of the BKMR model.**

| Variables | PIPs |
| --- | --- |
| DHBMA | 0.916 |
| 3HPMA | 0.8292 |
| MHBMA3 | 0.78564 |
| ATCA | 0.67916 |
| CYMA | 0.534 |
| 34MH | 0.50192 |
| AMCC | 0.4722 |
| AAMA | 0.46376 |
| PGA | 0.43364 |
| CEMA | 0.4132 |
| BMA | 0.3636 |
| HPM2 | 0.33892 |

Notes: Analysis incorporated adjustments for age, sex, race, education level, marital status, PIR, BMI, drinking and smoking status, diabetes, hypertension, and sedentary time. PIPs: posterior inclusion probabilities.
