# Supplementary material for: Exposure to volatile organic compounds increases the risk of sarcopenia: Insights into association and mechanism
Source: PLoS One. 2025 Oct 31;20(10):e0335660. doi: 10.1371/journal.pone.0335660 (PMC12578169; doi:10.1371/journal.pone.0335660)
Supplement: S1 Fig — (DOCX) [file pone.0335660.s004.docx]

**S1 Fig 1. Flowchart of the participants included in this study.**


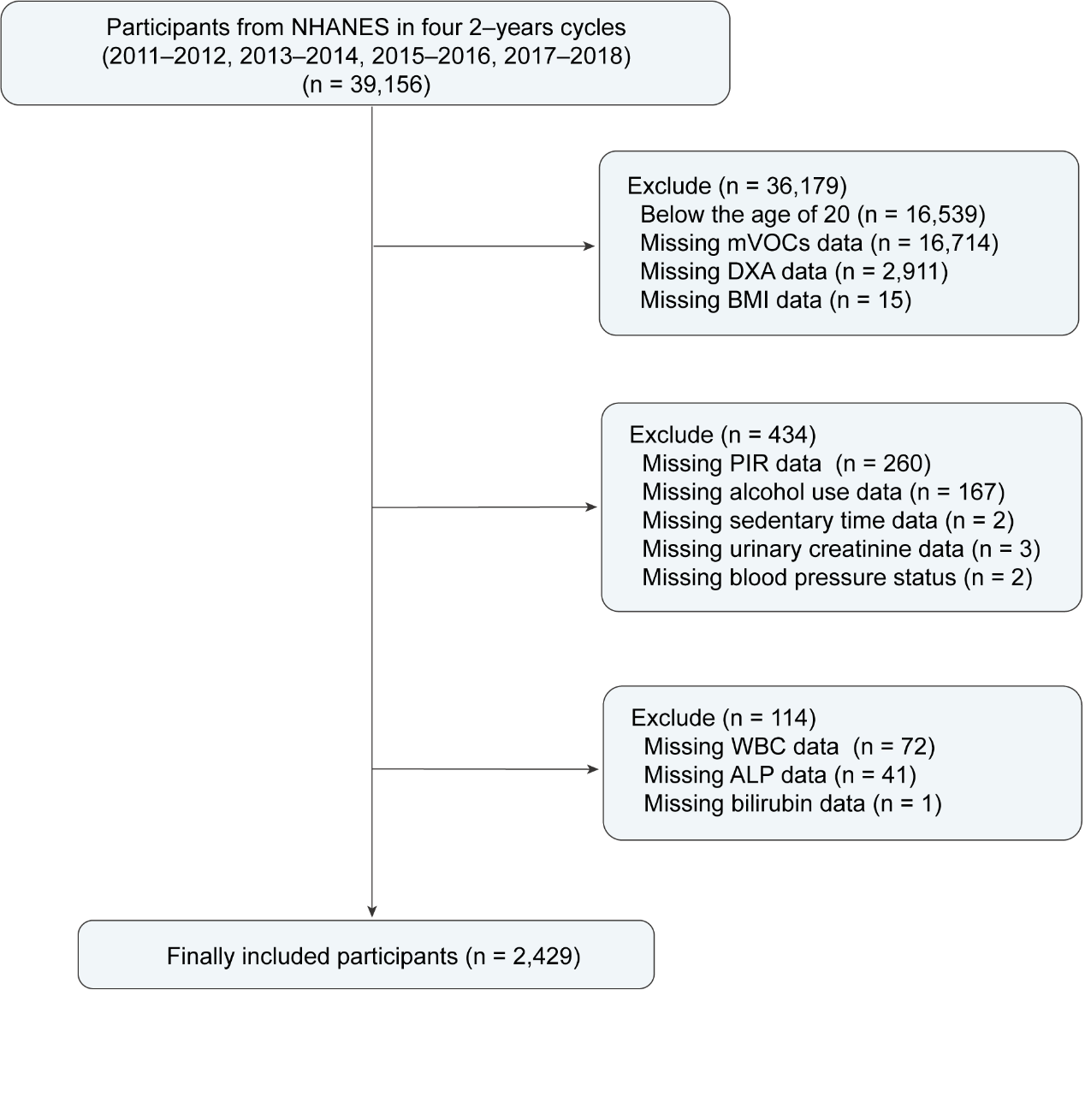


Notes: DXA: dual-energy X-ray absorptiometry, BMI: body mass index, PIR: family poverty income ratio, WBC: white blood cell, ALP: alkaline phosphatase.
