# Supplementary figures and images for: Exposure to volatile organic compounds increases the risk of sarcopenia: Insights into association and mechanism
Source: PLoS One. 2025 Oct 31;20(10):e0335660. doi: 10.1371/journal.pone.0335660 (PMC12578169; doi:10.1371/journal.pone.0335660)

**S1 Fig 2.** **Spearman correlation coefficients among the 16 mVOCs.**


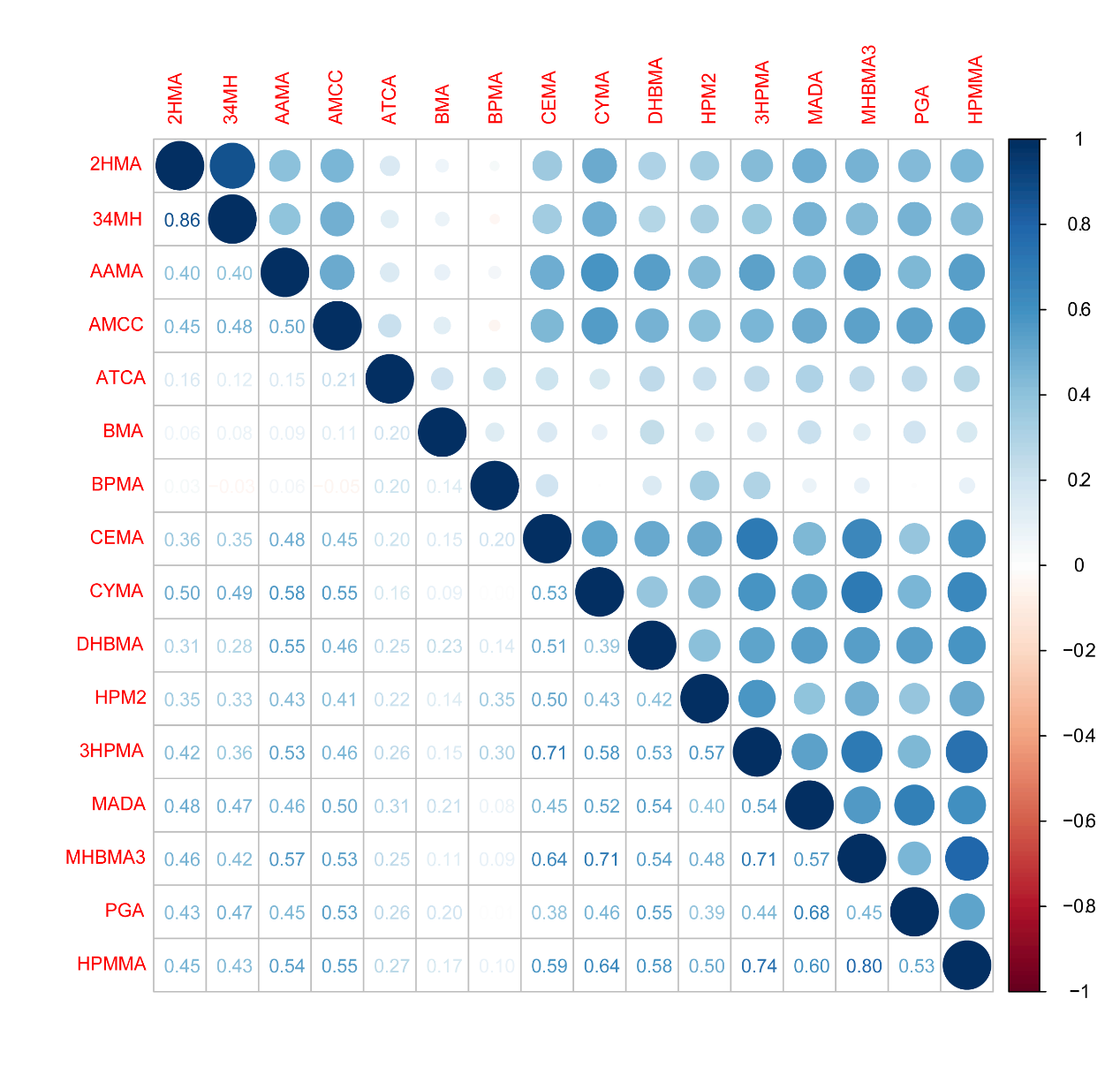

Supplement: S2 Fig — (DOCX) [file pone.0335660.s005.docx]
