# Supplementary material for: Exposure to volatile organic compounds increases the risk of sarcopenia: Insights into association and mechanism
Source: PLoS One. 2025 Oct 31;20(10):e0335660. doi: 10.1371/journal.pone.0335660 (PMC12578169; doi:10.1371/journal.pone.0335660)
Supplement: S3 Fig — (DOCX) [file pone.0335660.s006.docx]

**S1 Fig 3. Association between mVOCs and sarcopenia assessed by BKMR model.**

**(A)**

**
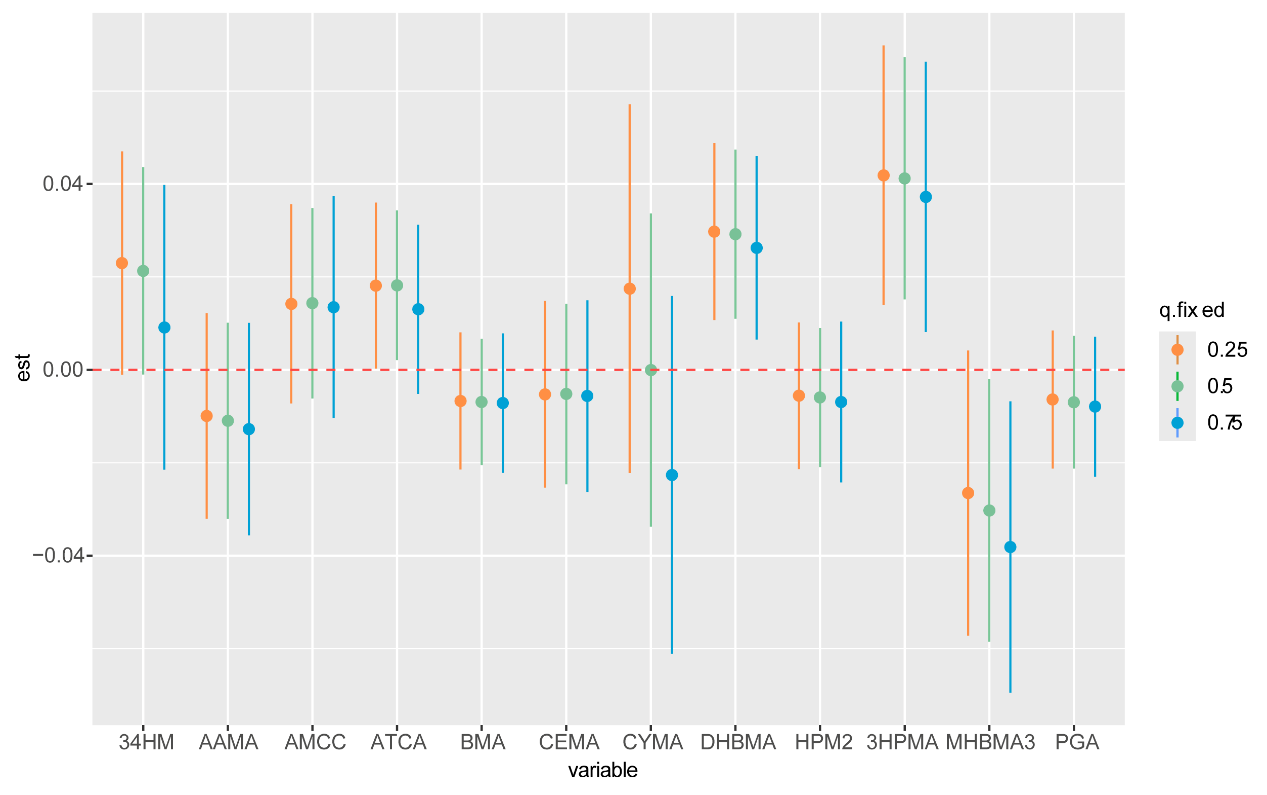
(B)**

**
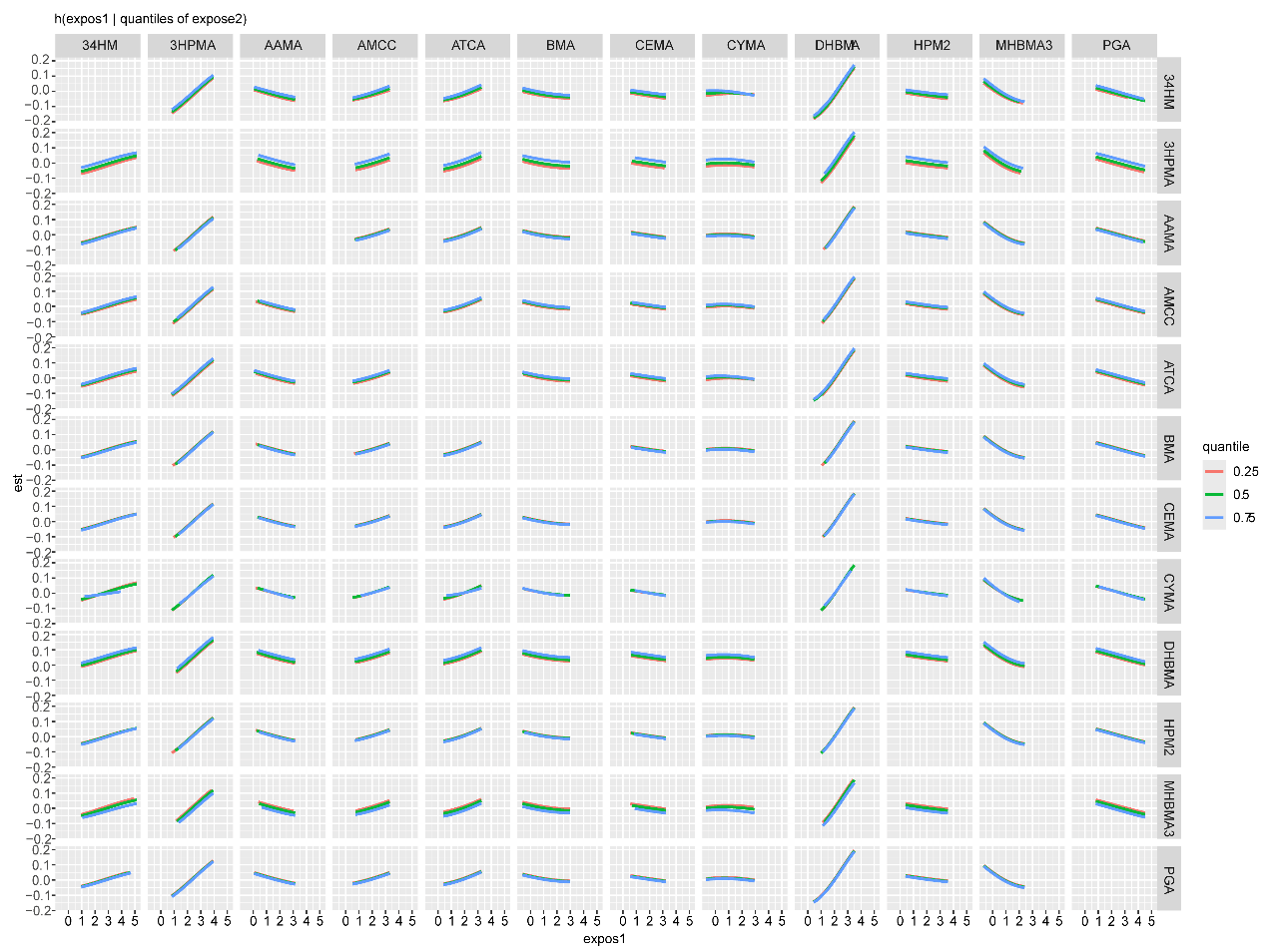
**

Notes: The analysis incorporated adjustments for age, sex, race, education level, marital status, PIR, BMI, drinking and smoking status, diabetes, hypertension, and sedentary time. (A) Univariate exposure–response functions between the screened mVOCs and sarcopenia. (B) Bivariate exposure–response functions between the screened mVOCs and sarcopenia.
