# Supplementary material for: Exposure to volatile organic compounds increases the risk of sarcopenia: Insights into association and mechanism
Source: PLoS One. 2025 Oct 31;20(10):e0335660. doi: 10.1371/journal.pone.0335660 (PMC12578169; doi:10.1371/journal.pone.0335660)
Supplement: S4 Fig — (DOCX) [file pone.0335660.s007.docx]

**S1 Fig 4. Mediation analysis of inflammatory and oxidative stress markers.**

**
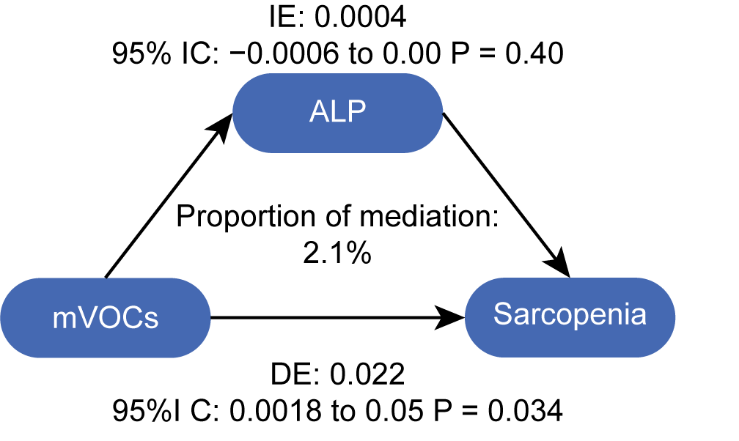
**
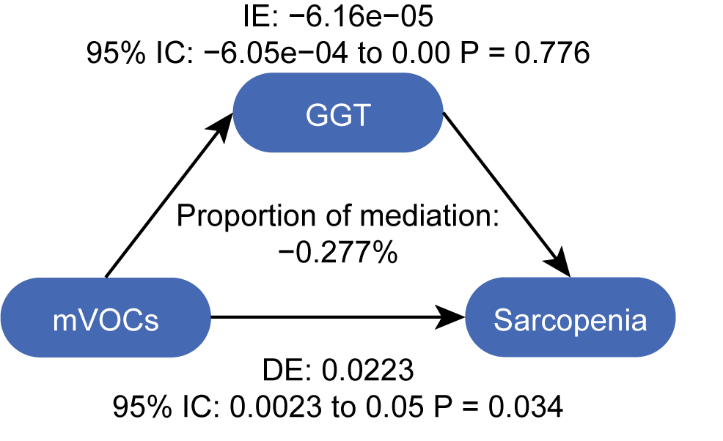


Notes: The analysis incorporated adjustments for age, sex, race, education level, marital status, PIR, BMI, drinking and smoking status, diabetes, hypertension, and sedentary time. ALP, alkaline phosphatase; GGT, gamma-glutamyltransferase; mVOCs, metabolites of volatile organic compounds; IE, indirect effect; DE, direct effect
