# Supplementary material for: Exposure to volatile organic compounds increases the risk of sarcopenia: Insights into association and mechanism
Source: PLoS One. 2025 Oct 31;20(10):e0335660. doi: 10.1371/journal.pone.0335660 (PMC12578169; doi:10.1371/journal.pone.0335660)
Supplement: S3 File — (DOCX) [file pone.0335660.s009.docx]

**Supplementary file S3**

**Exposure to volatile organic compounds increases the risk of sarcopenia:** **Insights into association and mechanism**

Maosheng Yang^1¶^, Hanbin Wang^1¶^, Wenlong Huang^1^, Yi Li^1*^

^1^Department of Joint Surgery and Sports Medicine, Shandong Provincial Hospital Affiliated to Shandong First Medical University, Jinan, 250000, China.

* Corresponding author:

E-mail: [liyivincent@126.com](mailto:liyivincent@126.com) (YL)

^¶^These authors also contributed equally to this work.

**Data extraction**

setwd("D:\\BaiduSyncdisk\\NHANES\\VOC\\1718")

VOCB1718<-read_xpt("D:\\BaiduSyncdisk\\NHANES\\VOC\\1718\\VOCWB_H.XPT")

write.xlsx(x=VOCB1718,file="D:\\BaiduSyncdisk\\NHANES\\VOC\\1718\\VOCB1718.xlsx")

write.csv(x=VOCB1718,file="D:\\BaiduSyncdisk\\NHANES\\VOC\\1718\\VOCB1718.csv",row.names = F)

library(readxl)

DX17181<- read_excel("D:\\BaiduSyncdisk\\NHANES\\VOC\\1718\\DX17181.xlsx")

library(plyr)

VOCdataall1718<-join_all(list(Activity1718,Creatinine1718,Alcohol1718,BIO1718,BloodCount1718,BP1718,Demographic1718,Diabetes1718,MCQ1718,VOC1718,Smoke1718,BMX1718,DX1718),by="SEQN",type="full")

write.xlsx(x=VOCdataall1718,file="D:\\BaiduSyncdisk\\NHANES\\VOC\\VOCdataall1718.xlsx")

write.csv(x=VOCdataall1718,file="D:\\BaiduSyncdisk\\NHANES\\VOC\\VOCdataall1718.csv",row.names = F)

VOCdataall1718T<-VOCdataall1718%>% select(SEQN,#SEQN

RIAGENDR,#SEX

RIDAGEYR,#AGE

RIDRETH1,# race/ethnicity

DMDEDUC2,# education level

INDFMPIR,# family poverty income ratio

DMDMARTL,# marital status 1Married，2Widowed，3Divorced，4Separated，5Never married，6Living with partner，77Refused，99Don't know

ALQ101,# drinking status

SMQ020,# smoking status

PAD680,# Minutes sedentary activity

BPQ020,# hypertension status

DIQ010,# diabetes status

BMXBMI,# Body Mass Index (kg/m**2)

DXDLALE,#Muscle mass of the left upper limbg

DXDLLLE,# Muscle mass of the left lower limbg

DXDRALE,# Muscle mass of the right upper limbg

DXDRLLE,# Muscle mass of the right lower limbg

URX2MH,# 2-methylhippuric acid (ng/mL)

URD2MHLC,# 2-methylhippuric acid comment code

URX34M,# 3-methipurc acd & 4-methipurc acd(ng/mL)

URD34MLC,# 3-methipurc acd & 4-methipurc acid comt

URXAAM,# N-ace-S-(2-carbamoylethyl)-L-cys(ng/mL)

URDAAMLC,# N-ace-S-(2-carbamoylethyl)-L-cys comt

URXAMC,# N-ace-S-(N-methlcarbamoyl)-L-cys(ng/mL)

URDAMCLC,# N-ace-S-(N-methlcarbamoyl)-L-cys comt

URXATC,# 2-amnothiazolne-4-carbxylic acid(ng/mL)

URDATCLC,# 2-amnothiazolne-4-carbxylic acid comt

URXBMA,# N-acetyl-S-(benzyl)-L-cysteine(ng/mL)

URDBMALC,# N-acetyl-S-(benzyl)-L-cysteine comt

URXBPM,# N-acetyl-S-(n-propyl)-L-cysteine(ng/mL)

URDBPMLC,# N-acetyl-S-(n-propyl)-L-cysteine comt

URXCEM,# N-acetyl-S-(2-carbxyethyl)-L-cys(ng/mL)

URDCEMLC,# N-acetyl-S-(2-carbxyethyl)-L-cys comt

URXCYM,# N-acetyl-S-(2-cyanoethyl)-L-cyst(ng/mL)

URDCYMLC,# N-acetyl-S-(2-cyanoethyl)-L-cyst comt

URXDHB,# N-ace-S-(3,4-dihidxybutl)-L-cys(ng/mL)

URDDHBLC,# N-ace-S-(3,4-dihidxybutl)-L-cys comt

URXGAM,# N-ac-S-(2-carbmo-2-hydxel)-L-cys(ng/mL)

URDGAMLC,# N-ac-S-(2-carbmo-2-hydxel)-L-cys comt

URXHEM,# N-ace-S-(2-hydroxyethyl)-L-cys(ng/mL)

URDHEMLC,# N-ace-S-(2-hydroxyethyl)-L-cys comt

URXHP2,# N-ace-S-(2-hydroxypropyl)-L-cys(ng/mL)

URDHP2LC,# N-ace-S-(2-hydroxypropyl)-L-cys comt

URXHPM,# N-ace-S-(3-hydroxypropyl)-L-cys(ng/mL)

URDHPMLC,# N-ace-S-(3-hydroxypropyl)-L-cys comt

URXMAD,# Mandelic acid(ng/mL)

URDMADLC,# Mandelic acid comment code

URXMB3,# N-A-S-(4-hydrxy-2-butenyl)-L-cys(ng/mL)

URDMB3LC,# N-A-S-(4-hydrxy-2-butenyl)-L-cys comt

URXPHE,# N-ace-S-(phenl-2-hydxyetl)-L-cys(ng/mL)

URDPHELC,# N-ace-S-(phenl-2-hydxyetl)-L-cys comt

URXPHG,# Phenylglyoxylic acid(ng/mL)

URDPHGLC,# Phenylglyoxylic acid comment code

URXPMM,# N-A-S-(3-hydrxprpl-1-metl)-L-cys(ng/mL)

URDPMMLC,# N-A-S-(3-hydrxprpl-1-metl)-L-cys comt

URXUCR,# Urinary creatinine

LBXWBCSI,# White blood cell count (1000 cells/uL)

LBXSAPSI,# alkaline phosphatase (U/L)

LBXSGTSI,# Gamma glutamyl transferase (U/L)

LBXSTB,# Total bilirubin (mg/dL)

LBDSTBSI)# Bilirubin, total (umol/L)

write.xlsx(x=VOCdataall1718T,file="D:\\BaiduSyncdisk\\NHANES\\VOC\\VOCdataall1718T.xlsx")

write.csv(x=VOCdataall1718T,file="D:\\BaiduSyncdisk\\NHANES\\VOC\\VOCdataall1718T.csv",row.names = F)

**Create baseline table:**

install.packages("tableone")

library(tableone)

library(readxl)

setwd("D:\\BaiduSyncdisk\\NHANES\\VOC")

VOCtotal1<- read_excel("D:\\BaiduSyncdisk\\NHANES\\VOC\\CrVOC数据（连续）.xlsx")

Table1<-CreateTableOne(data= VOCtotal1)

vars<-c("SEQN","RIAGENDR","RIDAGEYR","RIDRETH1","DMDEDUC2","INDFMPIR","DMDMARTL","ALQ101","SMQ020","PAD680","BPQ020","DIQ010","BMXBMI","DXDLALE","DXDLLLE","DXDRALE","DXDRLLE","URX2MH","URX34M","URXAAM","URXAMC","URXATC","URXBMA","URXBPM","URXCEM","URXCYM","URXDHB","URXHP2","URXHPM","URXMAD","URXMB3","URXPHG","URXPMM","URXUCR","LBXWBCSI","LBXSAPSI","LBXSGTSI","LBXSTB","LBDSTBSI","SP")

fvars<-c("RIAGENDR","RIDRETH1","DMDEDUC2","DMDMARTL","ALQ101","SMQ020","BPQ020","DIQ010","SP")

Table1T<- CreateTableOne(data= VOCtotal1,vars=vars,factorVars=fvars)

CreateTableOne(data= VOCtotal1,vars=vars,factorVars=fvars)

print(Table1T, showAllLevels=T)

Table1T<-print(Table1T,

showAllLevels=T,

nonnormal=c("RIDAGEYR","INDFMPIR","PAD680","BMXBMI","DXDLALE","DXDLLLE","DXDRALE","DXDRLLE","URX2MH","URX34M","URXAAM","URXAMC","URXATC","URXBMA","URXBPM","URXCEM","URXCYM","URXDHB","URXHP2","URXHPM","URXMAD","URXMB3","URXPHG","URXPMM","URXUCR","LBXWBCSI","LBXSAPSI","LBXSGTSI","LBXSTB","LBDSTBSI"))

write.csv(Table1T,file="Table1T.csv")

Table1TSP<- CreateTableOne(data= VOCtotal1,vars=vars,factorVars=fvars,strata="SP")

###全部展开显示

print(Table1TSP,

showAllLevels=T,

nonnormal=c("RIDAGEYR","INDFMPIR","PAD680","BMXBMI","DXDLALE","DXDLLLE","DXDRALE","DXDRLLE","URX2MH","URX34M","URXAAM","URXAMC","URXATC","URXBMA","URXBPM","URXCEM","URXCYM","URXDHB","URXHP2","URXHPM","URXMAD","URXMB3","URXPHG","URXPMM","URXUCR","LBXWBCSI","LBXSAPSI","LBXSGTSI","LBXSTB","LBDSTBSI"))

Table1TSP<- print(Table1TSP,

showAllLevels=T,

nonnormal=c("RIDAGEYR","INDFMPIR","PAD680","BMXBMI","DXDLALE","DXDLLLE","DXDRALE","DXDRLLE","URX2MH","URX34M","URXAAM","URXAMC","URXATC","URXBMA","URXBPM","URXCEM","URXCYM","URXDHB","URXHP2","URXHPM","URXMAD","URXMB3","URXPHG","URXPMM","URXUCR","LBXWBCSI","LBXSAPSI","LBXSGTSI","LBXSTB","LBDSTBSI"))

write.csv(Table1TSP,file="Table1TSP.csv")

**Logistic regression**

library(dplyr)

library(openxlsx)

library(caret)

library(readxl)

setwd("D:\\BaiduSyncdisk\\NHANES\\VOC\\2429")

data<- read_excel("D:\\BaiduSyncdisk\\NHANES\\VOC\\ 2429\\lgCrVOC2429.xlsx")

outcome_var <- "SP"

independent_vars <- c("URX2MH","URX34M","URXAAM","URXAMC","URXATC","URXBMA","URXBPM","URXCEM","URXCYM","URXDHB","URXHP2","URXHPM","URXMAD","URXMB3","URXPHG","URXPMM")

covariates <- c("RIAGENDR", "RIDRETH1", "DMDEDUC2", "DMDMARTL","ALQ101","SMQ020","BPQ020","DIQ010")

data[covariates] <- lapply(data[covariates], factor)

covariates<- c(covariates,"RIDAGEYR","INDFMPIR","BMXBMI","PAD680")

results <- list()

for (var in independent_vars) {

formula <- as.formula(paste(outcome_var, "~", var, "+", paste(covariates, collapse = "+")))

model <- glm(formula, family = binomial(link = "logit"), data = data)

or <- exp(coef(model))[var] # 提取OR值

ci <- exp(confint(model))[var, ] # 计算95%置信区间

p_value <- summary(model)$coefficients[var, 4] # 提取P值

results[[var]] <- list(or = or, ci = ci, p_value = p_value)

}

results_df <- do.call(rbind, lapply(names(results), function(var) {

data.frame(

Variable = var,

OR = results[[var]]$or,

CI_lower = results[[var]]$ci[1],

CI_upper = results[[var]]$ci[2],

P_value = results[[var]]$p_value

)

}))

write.xlsx(results_df, "VOCtotal1_results.xlsx")

**WQS Regression**

install.packages("gWQS")

library(gWQS)

filepath<-file.choose()

filepath

setwd("D:\\BaiduSyncdisk\\NHANES\\VOC")

wqs_data<-read.csv("D:\\BaiduSyncdisk\\NHANES\\VOC\\WQS-LASSO", sep =",", header = TRUE)

install.packages("corrplot")

library(corrplot)

head(wqs_data)

names(wqs_data)

cordata = wqs_data[,1:12]#12个自变量

cor.matrix<-cor(cordata,method = "spearman")#speraman

corrplot.mixed(cor.matrix, number.cex = 0.6)

corrplot.mixed(cor.matrix,lower = "number",upper = "circle",tl.pos = c("lt"),

diag = c("u"),bg = "white")

dput(names(wqs_data))

toxic_chems<-names(wqs_data)[1:12]

results<-gwqs(SP~wqs+RIAGENDR+RIDAGEYR+RIDRETH1+DMDEDUC2+INDFMPIR+DMDMARTL+ALQ101+SMQ020+PAD680+BPQ020+DIQ010+BMXBMI

,mix_name=toxic_chems,

data=wqs_data,q=4,validation=0.6,b=10000,b_pos=TRUE,b_constr=FALSE,family="binomial",seed=2023,plots=TRUE,tables=TRUE)

summary(results)

confint(results)#95%CI

summary(results)[["coefficients"]]

gwqs_scatterplot(results)

gwqs_summary_tab(results)

weight=as.data.frame(results$final_weights$mean_weight)

label= as.data.frame(results$final_weights$mix_name)

final_weight=cbind(label,weight)

View(final_weight)

final_weight=final_weight[order(final_weight$'results$final_weights$mean_weight'),]

names(final_weight)=c("Chems","weight")

final_weight$Chems=factor(final_weight$Chems,

levels=c("URX34M","URXAAM","URXAMC","URXATC","URXBMA","URXCEM","URXCYM","URXDHB","URXHP2","URXHPM","URXMB3","URXPHG"

))

final_weight$Chems=factor(final_weight$Chems,

levels=c("URXPHG","URXAAM","URXBMA","URXMB3","URXCEM","URXAMC","URXCYM","URXHP2","URXDHB","URXATC","URX34M","URXHPM"

))

install.packages('ggplot2')

library(ggplot2)

ggplot(final_weight,aes(x=Chems,y=weight,fill=Chems))+

geom_bar(stat="identity",color="black")+theme_bw()+

geom_hline(aes(yintercept=1/34),lty=2,color='grey',size=0.3)+

theme(axis.ticks = element_blank(),

axis.title = element_blank(),

plot.margin = unit(c(1,1,2,1),"lines"),

panel.grid.major = element_blank(),

panel.grid.minor = element_blank(),

panel.background = element_rect(fill="white",color="black"),

legend.position="none")+

coord_flip()

lifecycle::last_lifecycle_warnings()

**BKMR regression**

install.packages("bkmr")

install.packages("readxl")

install.packages("corrplot")

install.packages("ggplot2")

library("bkmr")

library("corrplot")

library("ggplot2")

library("readxl")

filepath<-file.choose()

filepath

setwd("D:\\BaiduSyncdisk\\NHANES\\VOC")

BKMRlg<-read.csv("D:\\BaiduSyncdisk\\NHANES\\VOC\\BKMRlg.csv",sep=",",header=TRUE)

dput(names(BKMRlg))

mixture<-as.matrix(BKMRlg[,1:19])

covariates<-as.matrix(BKMRlg[,20:31])

y<-BKMRlg$SP

knots50<-fields::cover.design(mixture,nd=50)$design

fit<-kmbayes(y=y,Z=mixture,X=covariates,iter=50,verbose=FALSE,varsel=TRUE,knots=knots50)

ExtractPIPs(fit)

pred.resp.univar<-PredictorResponseUnivar(fit=fit)

ggplot(pred.resp.univar,aes(z,est,ymin=est-1.96*se,ymax=est+1.96*se))+geom_smooth(stat="identity")+facet_wrap(~variable)+ylab("h(z)")

risks.overall<-OverallRiskSummaries(fit=fit,qs=seq(0.25,0.75,by=0.05),q.fixed=0.5)

ggplot(risks.overall,aes(quantile,est,ymin=est-1.96*sd,ymax=est+1.96*sd))+geom_pointrange()+geom_hline(yintercept=0,lty=2,col="red")

risks.singvar<-SingVarRiskSummaries(fit=fit,y=y,Z=mixture,X=covariates,qs.diff=c(0.25,0.75),q.fixed=c(0.25,0.50,0.75))

ggplot(risks.singvar,aes(variable,est,ymin=est-1.96*sd,ymax=est+1.96*sd,col=q.fixed))+geom_pointrange(position=position_dodge(width=0.75))+geom_hline(yintercept=0,lty=2,col="red")

expos.pairs = subset(data.frame(expand.grid(expos1=c(1,2,3,4,5,6,7,8,9,10,11,12),expos2=c(1,2,3,4,5,6,7,8,9,10,11,12))),expos1<expos2)

expos.pairs

pred.resp.bivar = PredictorResponseBivar(fit=fit,min.plot.dist = 0.5,z.pairs = expos.pairs)

ggplot(pred.resp.bivar,aes(z1,z2,fill=est))+

geom_raster()+

facet_grid(variable2~variable1)+

scale_fill_gradientn(colours = c('#0000FFFF','#FFFFFFFF','#FF0000FF'))+

xlab('expos1')+

ylab('expos2')+

ggtitle('h(expos1,expos2)')

pred.resp.bivar.levels <- PredictorResponseBivarLevels(

pred.resp.df = pred.resp.bivar,

Z = mixture, qs = c(0.25, 0.5, 0.75))

ggplot(pred.resp.bivar.levels, aes(z1, est)) +

geom_smooth(aes(col = quantile), stat = "identity") +

facet_grid(variable2 ~ variable1) +

ggtitle("h(expos1 | quantiles of expos2)") +

xlab("expos1")

geom_smooth()

**LASSO Regression**

library(glmnet)

library(readxl)

library(plyr)

library(caret)

library(corrplot)

library(ggplot2)

library(Hmisc)

library(openxlsx)

data<-read.csv("D:\\BaiduSyncdisk\\NHANES\\VOC\\LASSO回归162431.csv")

x<-as.matrix(data[,-c(1:2)])

y<-as.double(data$SP)

fit<-glmnet(x,y,family="binomial",nlambda = 100, alpha = 1)

print(fit)

plot(fit,xvar = "lambda")

set.seed(11)

lasso_fit<-cv.glmnet(x,y,family="binomial", alpha = 1, type.measure = "mse",nlambda=1000)

plot(lasso_fit)

print(lasso_fit)

lasso_best<- glmnet(x=x,y=y,alpha=1,lambda=lasso_fit$lambda.min)

coef(lasso_best)

coefficient<-coef(lasso_best,s=lasso_best$lambda.min)

coe<-coefficient@x

coe<-as.data.frame(coe)

Active_Index <-which(as.numeric(coefficient)!=0)

active_coefficients<-as.numeric(coefficient)[Active_Index]

variable<-rownames(coefficient)[Active_Index]

variable<-as.data.frame(variable)

variable<-cbind(variable,coe)

lambda.min <- lasso_fit$lambda.min

loglambda.min <- log(lambda.min)

cat("lambda.min:", lambda.min, "\n")

cat("loglambda.min:", loglambda.min, "\n")

**Mediating effects**

library(MASS)

library(Matrix)

library(mvtnorm)

library(sandwich)

library(mediation)

rm(list = ls())

data = read.csv(file = "D:\\BaiduSyncdisk\\NHANES\\VOC\\2429\\2429.csv")

set.seed(2024)

data$SP=as.factor(data$SP)

data$RIAGENDR=as.factor(data$RIAGENDR)

data$RIDRETH1=as.factor(data$RIDRETH1)

data$DMDMARTL=as.factor(data$DMDMARTL)

data$DMDEDUC2=as.factor(data$DMDEDUC2)

data$ALQ101=as.factor(data$ALQ101)

data$SMQ020=as.factor(data$SMQ020)

data$BPQ020=as.factor(data$BPQ020)

data$DIQ010=as.factor(data$DIQ010)

AAM", "URXAMC", "URXATC", "URXBMA", "URXCEM", "URXCYM", "URXDHB", "URXHP2", "URXHPM", "URXMB3", "URXPHG")

data[, vars_to_combine] <- scale(data[, vars_to_combine])

data$composite_treat <- rowMeans(data[, vars_to_combine])

M_composite <- lm(LBXSTB ~ composite_treat + RIAGENDR + RIDAGEYR + RIDRETH1 + DMDEDUC2 + INDFMPIR + DMDMARTL + ALQ101 + SMQ020 + PAD680 + DIQ010 + BMXBMI, data = data)

Y_composite <- glm(SP ~ LBXSTB + composite_treat + RIAGENDR + RIDAGEYR + RIDRETH1 + DMDEDUC2 + INDFMPIR + DMDMARTL + ALQ101 + SMQ020 + PAD680 + DIQ010 + BMXBMI, family = "binomial", data = data)

results_composite <- mediate(M_composite, Y_composite, treat = 'composite_treat', mediator = 'LBXSTB', boot = TRUE, sims = 1000)

summary(results_composite)

output <- capture.output(summary(results_composite))

output_file <- "LBXSTB.txt"

cat(output, file = output_file, sep = "\n")

**Forest map**

install.packages("forestploter")

library(grid)

library(forestploter)

library(readxl)

dt<-read.csv("D:\\BaiduSyncdisk\\NHANES\\VOC\\2429\\FOREST model 1.csv")

dt$" "<- paste(rep(" ",20),collapse = " ")

tm <- forest_theme(base_size = 9,

refline_col = "red",

arrow_type = "closed",

footnote_gp = gpar(col = "blue", cex = 0.6))

p <- forest(dt[c(1,7,8)],

est = dt$est,

lower = dt$low,

upper = dt$hi,

sizes = dt$se+0.1,

ci_column = 3,

ref_line = 1,

arrow_lab = c("p","m"),

xlim = c(0,4),

ticks_at = c(0.5, 1, 2, 3, 4),

footnote = "this is the demo data. Please ",

theme = tm)

plot(p)
